# Smarca4 maintains mitochondrial homeostasis and energy metabolism during cardiac development

**DOI:** 10.1007/s00018-026-06168-3

**Published:** 2026-03-07

**Authors:** Deung-Dae Park, Sujin Kim, Alena Boos, Yannik Andrasch, Leonie Krieg, Wolfgang Rottbauer, Steffen Just

**Affiliations:** 1 Molecular Cardiology, Department of Internal Medicine II, University of Ulm, Albert-Einstein-Allee 23, Ulm, 89081 Germany; 2 Department of Internal Medicine II, University of Ulm, Ulm, Germany

**Keywords:** SMARCA4, SWI/SNF complex, Heart, Skeletal muscle, Mitochondrial respiration, ATP

## Abstract

Mitochondrial metabolism is fundamental to cardiac and skeletal muscle function due to the high adenosine triphosphate (ATP) demand required for sustained contractility. Although mitochondrial dysfunction is central to metabolic myopathies, the epigenetic mechanisms regulating mitochondrial structure and function remain poorly defined. Here, we identify the SWI/SNF chromatin remodeling ATPase subunit Smarca4 as a critical regulator of mitochondrial homeostasis and cellular energy metabolism. Using a *smarca4a*-deficient zebrafish model (*smarca4a*^*a8−/−*^), we show that Smarca4 loss causes ventricular hypoplasia, pericardial edema, and disorganized skeletal muscle, leading to pronounced impairment of cardiac and muscular function. Heart-specific RNA-seq, ATAC-seq, and single-cell RNA-seq analyses revealed that Smarca4 deficiency reduces chromatin accessibility and suppresses the transcription of genes controlling mitochondrial biogenesis and oxidative phosphorylation. Consistently, high-resolution confocal imaging and Seahorse-based metabolic profiling demonstrated marked reductions in mitochondrial content, respiratory capacity, and ATP generation. AAV-mediated SMARCA4 knockdown in human cardiomyocytes and murine myotubes reproduced these mitochondrial defects. Collectively, these findings establish Smarca4 as a conserved chromatin remodeling factor linking nuclear regulation to mitochondrial energy homeostasis during vertebrate muscle development.

**Supplementary Information:**

The online version contains supplementary material available at 10.1007/s00018-026-06168-3.

## Introduction

Mitochondria are vital organelles responsible for generating cellular energy through oxidative phosphorylation (OXPHOS), producing adenosine triphosphate (ATP) to sustain essential biological processes. Beyond their role in energy metabolism, mitochondria regulate calcium homeostasis, reactive oxygen species (ROS) signaling, and apoptotic pathways. Mitochondrial dysfunction occurs when genetic mutations in either mitochondrial DNA (mtDNA) or nuclear DNA (ncDNA)-encoded mitochondrial proteins compromise ATP synthesis [1], leading to metabolic dysregulation, oxidative stress, and cellular impairment. Given the high energetic demands of tissues such as skeletal muscle and the myocardium, these organs are particularly susceptible to mitochondrial dysfunction. Mitochondrial myopathies represent a heterogeneous group of disorders characterized by impaired mitochondrial bioenergetics, manifesting clinically as progressive muscle weakness, exercise intolerance, and, in severe cases, cardiomyopathy and multi-organ failure [2, 3].

The SWI/SNF related BAF chromatin remodeling complex subunit ATPase 4 (SMARCA4) encodes the BRG1 protein, a core catalytic component of the SWI/SNF chromatin remodeling complex that modulates gene expression through nucleosome repositioning and chromatin reconstructing. By dynamically altering chromatin accessibility, SMARCA4 orchestrates diverse cellular processes, including cell differentiation, proliferation, and metabolic adaptation. In the cardiovascular system, SMARCA4 has emerged as a pivotal regulator of heart development, cardiac function, and gene expression. During cardiogenesis, SMARCA4 governs cardiac growth and lineage specification by regulating key cardiac transcription factors in both mice [4, 5] and zebrafish [6]. In the adult myocardium, continued SMARCA4 activity is essential for maintaining cardiac function, as its loss results in hypertrophic cardiomyopathy and impaired contractility in mice [4, 7]. Notably, recent single-cell transcriptomic analyses and studies of human cardiac specimens have revealed pathological dysregulation of SMARCA4 expression and activity in patients with hypertrophic and dilated cardiomyopathy as well as coronary heart disease [8, 9], further underscoring its indispensable role in maintaining cardiac homeostasis.

The *smarca4a*-deficient zebrafish mutant line, *smarca4a*^*a8−/−*^, was initially identified as exhibiting defects in retinal cell differentiation and disrupted cardiac development during embryogenesis [10]. Another *smarca4a* mutant strain (*brg1*^*s481*^) also displayed cardiac abnormalities, including cardiac hypoplasia and severe arrhythmia, in developing embryos [6]. These findings suggest that *smarca4a* plays a crucial role in cardiac growth and function, yet the underlying mechanisms remain largely unexplored. In this study, we demonstrate that loss of *smarca4a* impairs both cardiac morphogenesis and skeletal muscle organization, contributing to functional deficits in the heart and skeletal muscle of *smarca4a*^*a8−/−*^ mutants. Genome-wide profiling (ATAC-, RNA-, and scRNA-seq) of *smarca4a*^*a8−/−*^ hearts revealed that loss of *smarca4a* suppresses chromatin accessibility and transcription of genes essential for mitochondrial homeostasis and function. We confirmed that *smarca4a* depletion leads to a reduction in mitochondrial distribution within cardiomyocytes and skeletal muscle cells in vivo. Furthermore, metabolic profiling demonstrated that mitochondrial defects in *smarca4a*-deficient zebrafish result in impaired mitochondrial respiration. To extend these findings to mammalian systems, we investigated the role of SMARCA4 in mitochondrial homeostasis in human cardiomyocytes (AC16) and mouse skeletal muscle cells (C2C12) using AAV-mediated SMARCA4 ablation, which led to significant mitochondrial dysfunction. Collectively, these findings provide novel insights into the regulatory role of Smarca4/SMARCA4 in mitochondrial homeostasis and energy metabolism, highlighting its essential function in transcriptional control of mitochondrial maintenance as well as muscle development and function.

## Results

### Loss of *smarca4a* impairs the function of heart and skeletal muscle in developing zebrafish

**Fig. 1:**
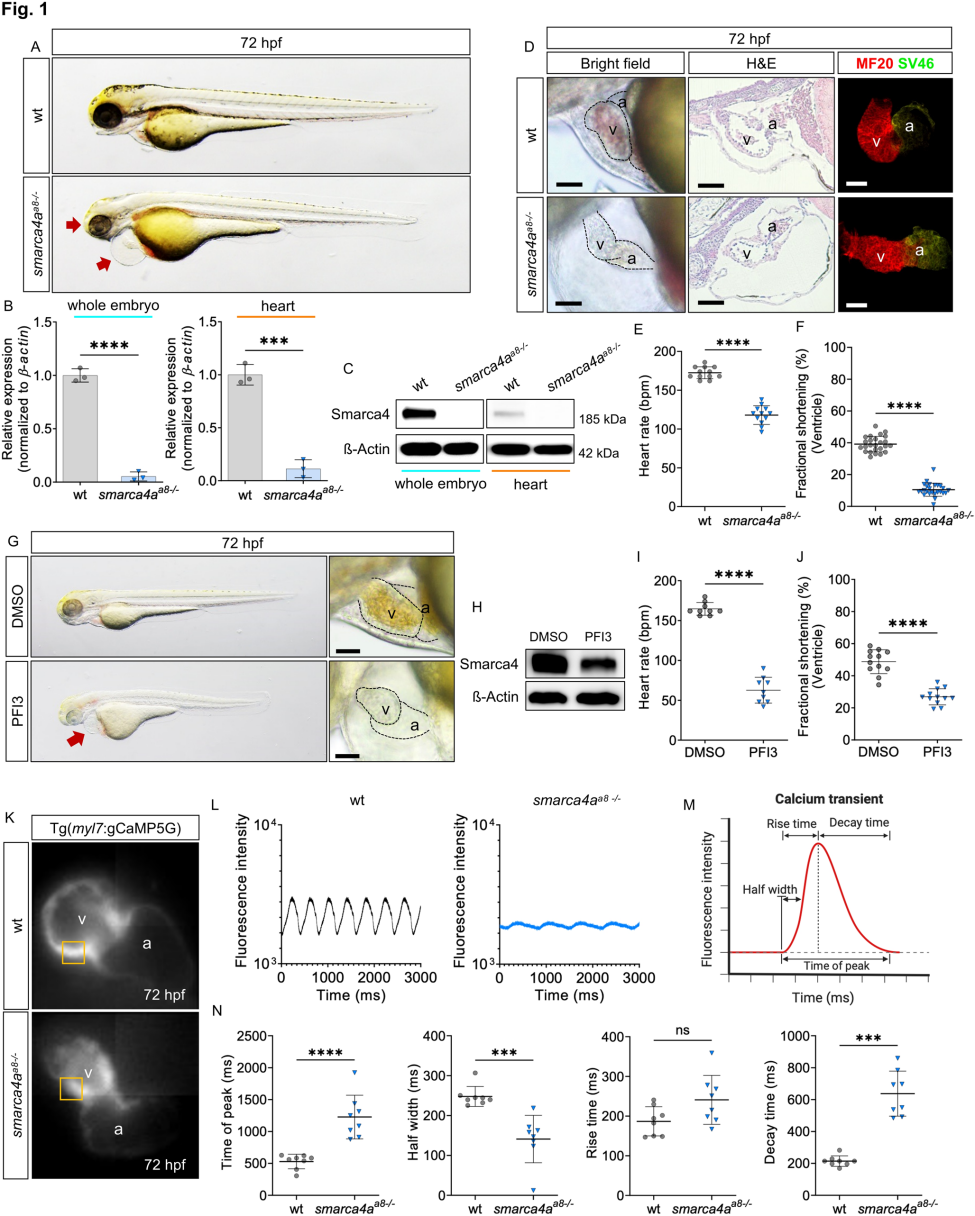
Loss of *smarca4* leads to cardiomyopathy in zebrafish embryonic heart. **A** Lateral view of wild-type (wt) and *smarca4* mutant (*smarca4a*^*a8−/−*^) zebrafish embryos at 72 hpf. The mutant embryo exhibits phenotypic defects in eyes and heart (red arrows). **B** Quantitative RT-PCR results of wt and *smarca4a*^*a8−/−*^ in whole embryo or heart (whole embryo: wt: 1.0 ± 0.06, *smarca4a*^*a8−/−*^: 0.05 ± 0.04; heart: wt: 1.0 ± 0.10, *smarca4a*^*a8−/−*^: 0.11 ± 0.09, SD, *n* = 3, ****p < 0.0001). Transcription level of *smarca4* is decreased in both tissues compared to wt. **C** Western blot assay showing the absence of Smarca4 protein in whole embryo or heart tissue of wt and *smarca4a*^*a8−/−*^ at 72 hpf. Protein level of Smarca4 is decreased in both tissues compared to wt. **D** Bright field images, sagittal histological sections (H&E: Hematoxylin & Eosin staining), or MF20/S46 staining of wt and *smarca4* mutant hearts at 72 hpf (scale bar: 50 μm). *Smarca4a*^*a8−/−*^ displayed cardiac edema and defective cardiac looping, but cardiac chamber differentiation and specification were not affected. v=ventricle, a=atrium. **E** Heart rate of wt and *smarca4a*^*a8−/−*^ embryos at 72 hpf (bpm: beats per minute, wt: 172.5 ± 7.73, *smarca4a*^*a8−/−*^: 118.0 ± 12.09, SD, *n* = 12, ****p < 0.0001). **F** Quantification of measured ventricular contractility in wt and *samrca4a*^*a8−/−*^ hearts at 72 hpf (wt: 39.16 ± 4.87, *smarca4a*^*a8−/−*^: 10.45 ± 4.11, SD, *n* = 25, ****p < 0.0001). Cardiac function of *smarca4*-deficient embryo was severely impaired compared to wt embryos. **G** Lateral view of DMSO or PFI3 treated zebrafish embryos at 72 hpf (scale bar: 50 μm). **H** Western blot bands showing Smarca4 protein levels in compound-mediated Smarca4 inhibition in zebrafish embryos at 72 hpf. Beta-actin (ß-Actin) is used as house-keeping protein. **I** Heart beating frequency of DMSO or PFI3 treated embryos at 72 hpf (wt: 164.57 ± 8.00, *smarca4a*^*a8−/−*^: 62.67 ± 16.19, SD, *n* = 9, ****p < 0.0001). **J** Fractional shortening analysis of DMSO or PFI3 treated zebrafish embryonic ventricle (wt: 48.76 ± 7.46, *smarca4a*^*a8−/−*^: 26.90 ± 4.99, SD, *n* = 12, ****p < 0.0001). Pharmacological inhibition of Smarca4 leads to cardiac dysfunction in zebrafish embryonic heart. **K** Confocal images showing Ca^2+^ in the hearts of wt and *samrca4a*^*a8−/−*^, crossed with Tg(*myl7*:gCaMP5G) visualizing heart-specific GFP-fused calcium binding protein (calmodulin). **L** Calcium transient of wt and *smarca4* mutant ventricles at 72 hpf. Calcium transient is severely blunted in the ventricle of *smarca4a*^*a8−/−*^ embryos. **M** Illustration of parameters for analyzing the calcium transient. **N** Quantification of calcium transients (time of peak, half width, rise time, and decay time) in the ventricle of wt and *smarca4* mutant embryos at 72 hpf (Time of peak: wt: 528.88 ± 113.71, *smarca4a*^*a8−/−*^: 1277.3 ± 342.40; Half width: wt: 247.91 ± 25.04, *smarca4a*^*a8−/−*^: 141.03 ± 59.37; Rise time: wt: 186.77 ± 36.76, *smarca4a*^*a8−/−*^: 240.91 ± 61.65; Decay time: wt: 214.54 ± 32.76, *smarca4a*^*a8−/−*^: 637.12 ± 141.29, SD, *n* = 8, ns: *p* > 0.05, ***p < 0.001, ****p < 0.0001).

The phenotypic characteristics of *smarca4a*-deficient (*smarca4a*^*a8−/−*^) zebrafish embryos include defective eye development and pericardial edema accompanied by abnormal heart morphology during developmental stages (48, 72, and 96 h post-fertilization (hpf)). (Fig. S1A; Fig. 1A), consistent with previous observations [6, 10]. The *smarca4a*^*a8−/−*^ mutation was identified as a point mutation in *smarca4a*, resulting in a premature stop codon that leads to the truncation of functional domains [10]. To determine whether this point mutation affects transcriptional or translational expression of Smarca4, which has not been previously investigated, we performed quantitative RT-PCR (qRT-PCR) and Western blot analysis using whole embryos and heart tissues of *smarca4a*^*a8−/−*^ mutants at 72 hpf (Fig. 1B-C). Both *smarca4a* mRNA expression and Smarca4 protein levels were significantly reduced in whole embryos and heart tissues, consistent with decreased transcript abundance due to impaired transcription and/or nonsense-mediated mRNA decay. Next, we characterized *smarca4a*^*a8−/−*^ mutant embryos to further investigate morphological and functional cardiac defects. At 72 hpf, mutant embryos exhibited cardiac phenotypes such as pericardial edema, blood congestion, and reduced heart chamber size, while chamber specification remained unaffected (Fig. 1D). To substantiate if deformation of cardiac chambers, especially the ventricle, in *smarca4a*^*a8−/−*^ results from decreased cardiomyocyte numbers, we counted ventricular cardiomyocytes using wt or *smarca4a*^*a8−/−*^ crossed with Tg(*myl7*:dsred.nls), showing nucleic expression of cardiomyocyte in red fluorescence. The number of cardiomyocytes was significantly decreased in the ventricle of *smarca4a*^*a8−/−*^ compared to wt at 72 hpf (Fig. S2A). Furthermore, EdU incorporation assay and phosphorylated histone 3 (pH3) immunofluorescent (IF) staining revealed that proliferative capacity of cardiomyocytes was significantly reduced by loss of Smarca4 in developing zebrafish hearts (Fig. S2B-E). To determine whether cardiac function was also impaired in *smarca4a*^*a8−/−*^ mutants, we measured heart rate and fractional shortening of embryonic hearts at 72 hpf. Quantitative analyses revealed a significant reduction in both heart rate and contractility in *smarca4a*^*a8−/−*^ embryos compared to wild-type (wt) controls. To further validate the causal relationship between Smarca4 loss and cardiomyopathy, we inhibited Smarca4 using antisense oligonucleotides (morpholino: MO) and a Smarca4 bromodomain inhibitor (PFI3) (Fig. S3B, 1H). Both *smarca4a* MO injection and PFI3 treatment resulted in cardiac morphological (Fig. S3A, 1G) and functional (Fig. S3C-D, 1I-J) defects similar to those observed in *smarca4a*^*a8−/−*^ mutants, compared to their respective controls (control MO or DMSO) at 72 hpf. Notably, PFI3 treatment was associated with reduced Smarca4 protein levels, as assessed by Western blot analysis (Fig. 1H). Given that Ca²⁺ dysregulation is a well-established consequence of contractile dysfunction in cardiac diseases such as cardiomyopathy [11], we assessed calcium transients by imaging embryonic hearts of wt and *smarca4a*^*a8−/−*^ mutants crossed with Tg(*myl7*:gCaMP5G) [12]. Calcium transients in the ventricle, atrioventricular (AV) canal, and atrium were significantly blunted in *smarca4a*^*a8−/−*^ mutant hearts compared to wt hearts (Fig. 1K-L, S4A-B). Quantitative analysis of calcium transient parameters (Fig. 1M) revealed prolonged time to peak and delayed decay time in *smarca4a*^*a8−/−*^ hearts compared to wt at 72 hpf (Fig. 1N, S4C-D), demonstrating that loss of *smarca4a* impairs cardiac function in zebrafish embryonic heart.

In addition to cardiac abnormalities, the *smarca4a*^*a8−/−*^ embryo exhibit skeletal muscle defects at 72 hpf (Fig. S5A). Birefringence analysis revealed a significant reduction of birefringence intensity in *smarca4a*^*a8−/−*^ embryos compared to wt (Fig. S5B), indicating disrupted skeletal muscle organization. To determine whether the skeletal muscle abnormalities in *smarca4a*^*a8−/−*^ embryos results in functional impairments, we assessed motility using a touch response assay. As shown in Fig. S5C-D, *smarca4a*-deficient embryos exhibited severely impaired motility compared to wt siblings at 72 hpf. To further investigate if loss of *smarca4a* affects skeletal muscle structure or organization, we performed IF staining using Tropomyosin, F59 (anti-myosin heavy chain), and F310 (anti-myosin light chain), which label slow or fast muscle fibers, respectively. While striated slow and fast muscle fibers developed comparably between wt and *smarca4a*^*a8−/−*^ embryos at 72 hpf, all muscle fibers in the *smarca4a* mutants exhibited disorganized architecture compared to wt (Fig. S5E). Furthermore, both MO-mediated and pharmacological inhibition of Smarca4 resulted in skeletal muscle structural disorganization (Fig. S6A-C) as well as significant loss of motility in zebrafish embryos at 72 hpf (Fig. S6D-G). Similarly, Smarca4 inhibition further confirmed disorganization of skeletal muscle in zebrafish embryos (Fig. S6H-I). Collectively, these findings demonstrate that loss of Smarca4 leads to myopathy affecting both cardiac and skeletal muscle in developing zebrafish embryos.

### *Smarca4a* regulates chromatin accessibility and transcription of mitochondria-related genes

**Fig. 2:**
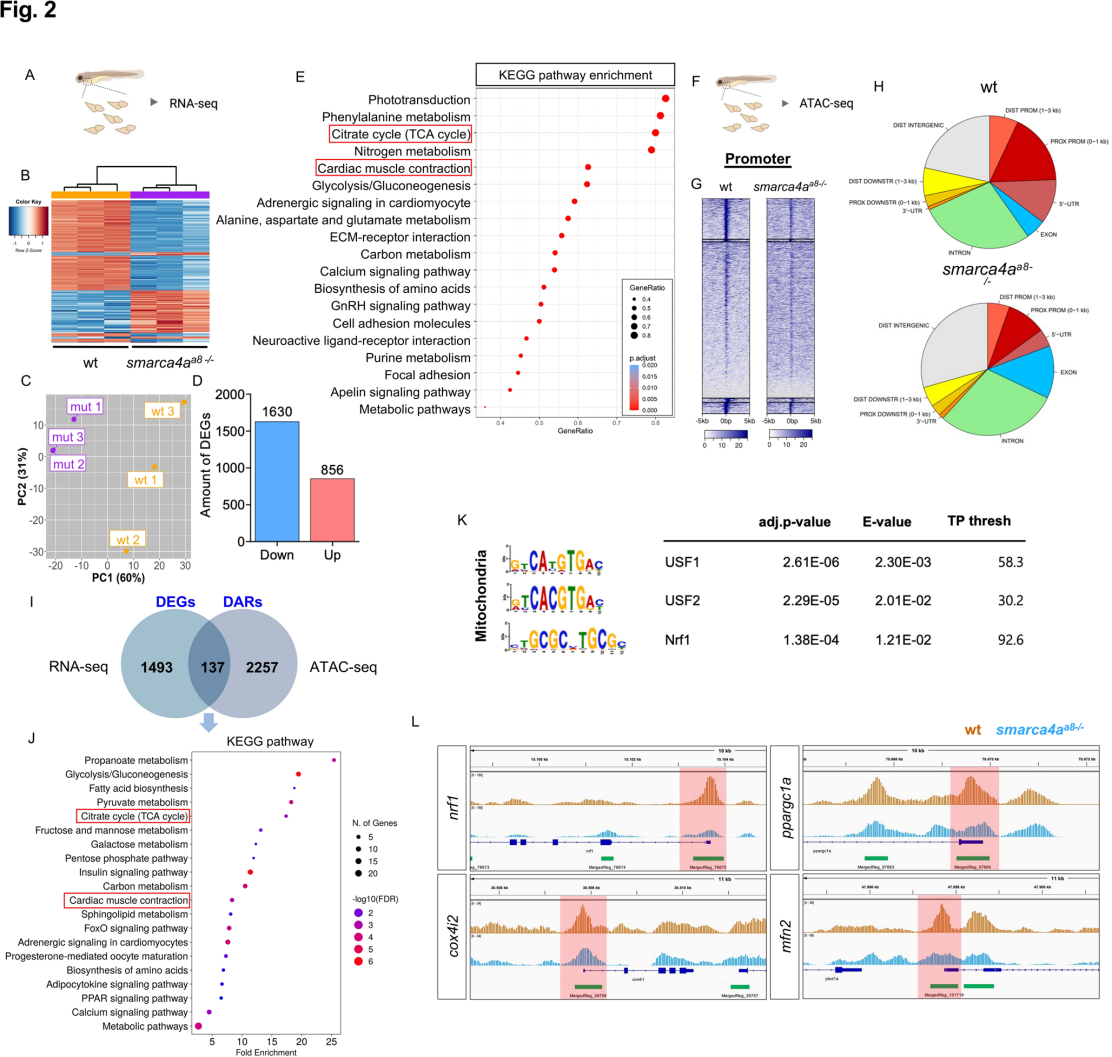
Loss of *smarca4* reduces transcription and chromatin accessibility of mitochondria-related genes in developing hearts. **A** Schematic workflow illustrating heart-specific RNA sequencing in zebrafish embryos. **B** Heatmap of the top 500 most varied genes in wt and *smarca4a*^*a8−/−*^. **C** PCA plots of RNA-seq in wt and *smarca4a* mutant embryos. mut=mutant. **D** Differentially expressed genes (DEGs) analysis of heart-specific RNA sequencing reveals 1630 down- and 856 upregulated genes in *smarca4a*^*a8−/−*^ (|log_2_(fold change: FC)| > 1, adjusted p-value < 0.05). **E** KEGG pathway enrichment analysis of suppressed genes in *smarca4a*^*a8−/−*^ shows the pathways of *citrate cycle (TCA cycle)* and *cardiac muscle contraction*. **F** Schematic workflow illustrating heart-specific ATAC sequencing in zebrafish embryos. **G** Heatmap visualization of ATAC-seq representing open chromatin sites on promoter regions in the hearts of wt and *smarca4a*^*a8−/−*^. **H** Pie charts of genomic features describing the location relative to genomic annotation in wt and *smarca4a*^*a8−/−*^. The annotated peaks in the proximal promoter site (PROX PROM (0–1 kb): red) are reduced in *smarca4a*^*a8−/−*^. **I** Venn diagram showing the overlap between downregulated DEGs (log_2_FC ≤ -1) and downregulated differentially accessible regions (DARs; merged peaks ≥ 100, log_2_FC ≤ -1, adj.p-value > 0.05). The light blue circle represents down regulated 1,630 DEGs unique to RNA-seq analysis, and the dark blue circle represents 2,394 DAR-associated genes unique to ATAC-seq analysis. The overlap contains 137 genes that are both differentially expressed and associated with differentially accessible chromatin regions. **J** The subsequent integrative analysis of KEGG pathway enrichment using overlapping 137 genes shows the pathways of *citrate cycle (TCA cycle)* and *cardiac muscle contraction*. **K** Sequence motifs enriched in the promoter regions of genes overlapping between downregulated DEGs and DARs. Motif enrichment analysis identified three highly enriched consensus sequences corrsponding to potential USF1-, USF2-, and Nrf1-binding motifs, suggesting that the overlapping DEG–DAR gene set is enriched for transcriptional regulators directly relevant to mitochondrial function. **L** Integrative Genomics Viewer tracks displaying chromatin accessibility of *nrf1*, *cox4i2*, *ppargc1a*, and *mfn2*. The light red boxes highlight merged peak regions in promotor regions. The peaks of the genes in proximal promoter regions are decreased in *smarca4a*^*a8−/−*^ compared to wt.

Smarca4 is recognized as both a core subunit and a functional regulator of the SWI/SNF chromatin remodeling complex, which modulates chromatin accessibility through nucleosome remodeling, thereby influencing transcriptional regulation [13]. To determine whether the loss of *smarca4a* alters the transcriptional landscape in zebrafish embryos, we performed RNA sequencing (RNA-seq) using wt and *smarca4a*^*a8−/−*^ embryonic hearts at 72 hpf (Fig. 2A-C). Transcriptomic analysis identified 1630 downregulated and 856 upregulated differentially expressed genes (DEGs) in *smarca4a*^*a8−/−*^ embryos (Fig. 2D). To elucidate transcription factors or biological pathways relevant to cardiac and skeletal muscle dysfunction, we clustered the downregulated DEGs using Gene Ontology (GO) terms and KEGG pathway analysis (Fig. S7A, 2E). GO-term enrichment revealed *skeletal muscle contraction*, *cardiac muscle contraction*, and *calcium ion transport*, aligning with our phenotypic findings in zebrafish embryos. Notably, we also identified GO terms associated with energy metabolism, including *ATP generation from ADP*, *ATP biosynthesis*, and *ATP metabolic processes*. Furthermore, KEGG pathway analysis demonstrated a significant downregulation of the *citrate cycle (TCA cycle)* and *cardiac muscle contraction* in *smarca4a*-deficient embryonic hearts. Next, we performed an Assay for Transposase-Accessible Chromatin coupled with high-throughput sequencing (ATAC-Seq) followed by bioinformatic analysis to examine genome-wide open chromatin profiles in *smarca4a*^*a8−/−*^ hearts (Fig. 2F). Compared to wt hearts, *smarca4a*^*8a−/−*^ exhibited a significant reduction in chromatin accessibility within transcription start sites (TSS: promoter regions) (Fig. 2G-H) (Proportion of intervals within the promoter region (0–1 kb)/total: wt: 20.27%, *smarca4a*^*a8−/−*^: 10.53%). To further elucidate the transcriptional regulatory mechanisms underlying the altered gene expression profile in *smarca4a*^*8a−/−*^ embryonic hearts, we integrated RNA-seq and ATAC-seq datasets to identify DEGs with concomitant changes in chromatin accessibility. Integrative analysis of suppressed DEGs and suppressed differentially accessible regions (DARs) revealed 137 genes common to both DEGs and DARs (Fig. 2I). KEGG pathway enrichment of these overlapping genes demonstrated significant representation of metabolic and contractile pathways including *citrate cycle (TCA cycle)* and *cardiac muscle contraction* (Fig. 2J). Motif enrichment analysis of DARs associated with these suppressed genes identified significantly enriched binding motifs of upstream stimulatory factor (USF) family members (USF1/USF2) and Nrf1 (Fig. 2K). USF1/USF2 and Nrf1 motifs are known to regulate oxidative phosphorylation [14], mitochondrial homeostasis [15], and mitochondrial biogenesis/function [16–18]. Thus, reduced accessibilities at these USFs/NRF1 motifs in *samrca4a*-deficient hearts suggest dysfunction of mitochondria-related genes, thereby exacerbating contractile deficits via impaired energy homeostasis. Given the enrichment of Nrf1-binding motifs within DARs associated with downregulated metabolic pathways, we examined chromatin accessibility at *nrf1*, *cox4i2*, *ppargc1a*, and *mfn2* loci in Smarca4-deficient zebrafish hearts. These genes are key regulators of mitochondrial DNA transcription [19], oxidative phosphorylation (OXPHOS**)** [20–22], or mitochondrial dynamics [23] (Fig. 2L), and their reduced accessibility supports impaired mitochondrial gene regulation upon Smarca4 loss. Representative genome browser tracks showed markedly reduced chromatin accessibility at the promoter regions of key mitochondria-related genes, implying defective transcription of mitochondria-related genes in *smarca4a*-deficient hearts.

**Fig. 3:**
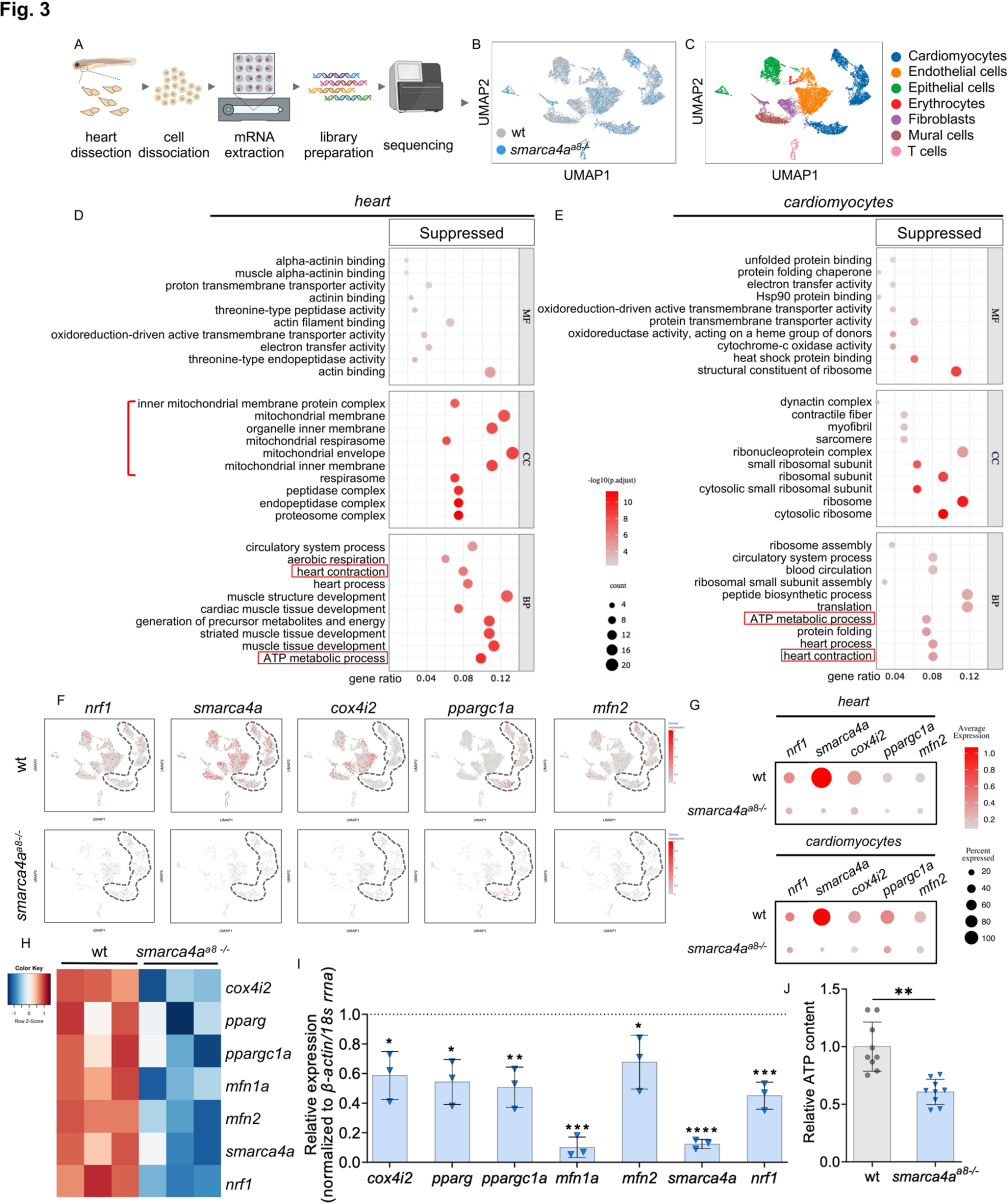
Transcriptional expression of mitochondria-related genes is suppressed in *smarca4a*^*a8−/−*^ heart and cardiomyocyte. **A** Schematic illustration of experimental design of single cell sequencing (scRNA-seq) analysis. **B-C** Integrated UMAP projections showing wt and *smarca4a*^*a8−/−*^ cells from dissected hearts (B) or depicting cell type clusters consisting of the embryonic hearts of wt and *smarca4a*^*a8−/−*^ (C) at 72 hpf. **D-E** GO-term enrichment analysis of suppressed DEGs in hearts (D) or cardiomyocytes € of wt and *smarca4a*^*a8−/−*^ (Log_2_FC < -0.5, p-value < 0.05). MF=molecular function, CC=cellular component, BP=biological process. **F** Gene expression plots expressing the indicated genes (*nrf1*, *smarca4a*, *cox4i2*, *ppargc1a*, and *mfn2*) colored red, and its relative intensity indicates relative expression levels. Gray dashed lines indicate cardiomyocytes cluster. **G** Dot plots displaying relative expression of *nrf1*, *smarca4a*, *cox4i2*, *ppargc1a*, and *mfn2*, which are reduced in the heart or cardiomyocytes of *smarca4a*^*a8−/−*^ compared to wt. **H** Visualization of wt and *smarca4a*^*a8−/−*^ heart-specific RNA-seq data showing mitochondria-related genes (*cox4i2*, *pparg*, *ppargc1a*, *mfn1a*, *mfn2*, and *nrf1*) and *smarca4a* by heatmap. **I** Quantitative RT-PCR results showing decreased mRNA expression of mitochondria-related genes (*cox4i2*, *pparg*, *ppargc1a*, *mfn1a*, *mfn2*, and *nrf1*) and *smarca4a* in *smarca4a*^*a8−/−*^ embryos compared to wt (*smarca4a*^*a8−/−*^: *cox4i2*: 0.59 ± 0.16, *pparg*: 0.54 ± 0.15, *ppargc1a*: 0.51 ± 0.14, *mfn1a*: 0.1 ± 0.07, *mfn2*: 0.68 ± 0.18, *smarca4a*: 0.12 ± 0.03, *nrf1*: 0.45 ± 0.09, SD, *n* = 3, *p < 0.05, **p < 0.01, ***p < 0.001, ****p < 0.0001). **J** Relative ATP content in wt and *smarca4a* mutant embryos (wt: 1.00 ± 0.12, *smarca4a*^*a8−/−*^: 0.61 ± 0.11, SD, *n* = 10, *N* = 9, **p < 0.01). ATP level of *smarca4a*^*a8−/−*^ is significantly decreased compared to wt.

To assess the cardiac-specific transcriptional consequences of *smarca4a* deficiency, we performed single-cell RNA-seq (scRNA-seq) and transcriptomic profiling at single-cell resolution using dissected hearts from wt and *smarca4a*^*a8−/−*^ at 72 hpf (Fig. 3A-C). Differential expression analysis revealed a significant downregulation of genes associated with mitochondrial function and energy metabolism (Fig. 3D-E). Gene ontology (GO) enrichment of suppressed transcripts identified cellular component (CC) terms related to mitochondria and the biological process (BP) GO term “*ATP metabolic process*” across multiple cardiac cell types (cardiomyocytes, endothelial cell, epithelial cells, erythrocytes, fibroblasts, mural cells, T cells) (Fig. 3D). To elucidate the impact of *smarca4a* loss on energy metabolic gene regulation, we analyzed the expression of *nrf1*, *smarca4a*, *cox4i2*, *ppargc1a*, and *mfn2* across all cell populations within *smarca4a*-deficient hearts. Compared to wt embryos, transcript levels of these genes were consistently reduced (Fig. 3F-G). Cardiomyocyte-specific transcriptomic profiling further demonstrated significant suppression of mitochondria-related gene expression, including *nrf1*, *cox4i2*, *ppargc1a*, and *mfn2* (Fig. 3F-G), as well as enrichment of the GO term “*ATP metabolic process*,” indicating impaired bioenergetic function in *smarca4a*-deficient cardiomyocytes (Fig. 3E). To further investigate transcriptional expression of genes involved in mitochondrial homeostasis, we assessed heart-specific RNA-seq and found that *cox4i2*, *pparg*, *ppargc1a*, *mfn1a*, *mfn2*, and *nrf1* were significantly downregulated in *smarca4a* mutant embryonic hearts (Fig. 3H). Additionally, *smarca4a* expression was significantly reduced in *smarca4a*^*a8−/−*^ hearts, further supporting the reliability of the transcriptional profiles defined in wt and *smarca4a*^*a8−/−*^. The downregulation of these genes in the *smarca4a*^*a8−/−*^ were confirmed via qRT-PCR (Fig. 3I), mirroring the heart-specific RNA-seq, ATAC-seq, and scRNA-seq findings. These results underlie that loss of *smarca4a* leads to decreased chromatin accessibility, thereby suppressing the transcription of genes essential for mitochondrial homeostasis and function. To determine whether the suppression of these genes correlates with a reduction in ATP production, we measured relative ATP levels in wt and *smarca4a* mutant embryos. Notably, ATP levels were significantly decreased in *smarca4a*-deficient embryos compared to wt controls (Fig. 3J), suggesting that mitochondrial function is impaired due to the loss of *smarca4a*.

### *Smarca4a* deficiency leads to structural damages and functional defects in mitochondria

**Fig. 4:**
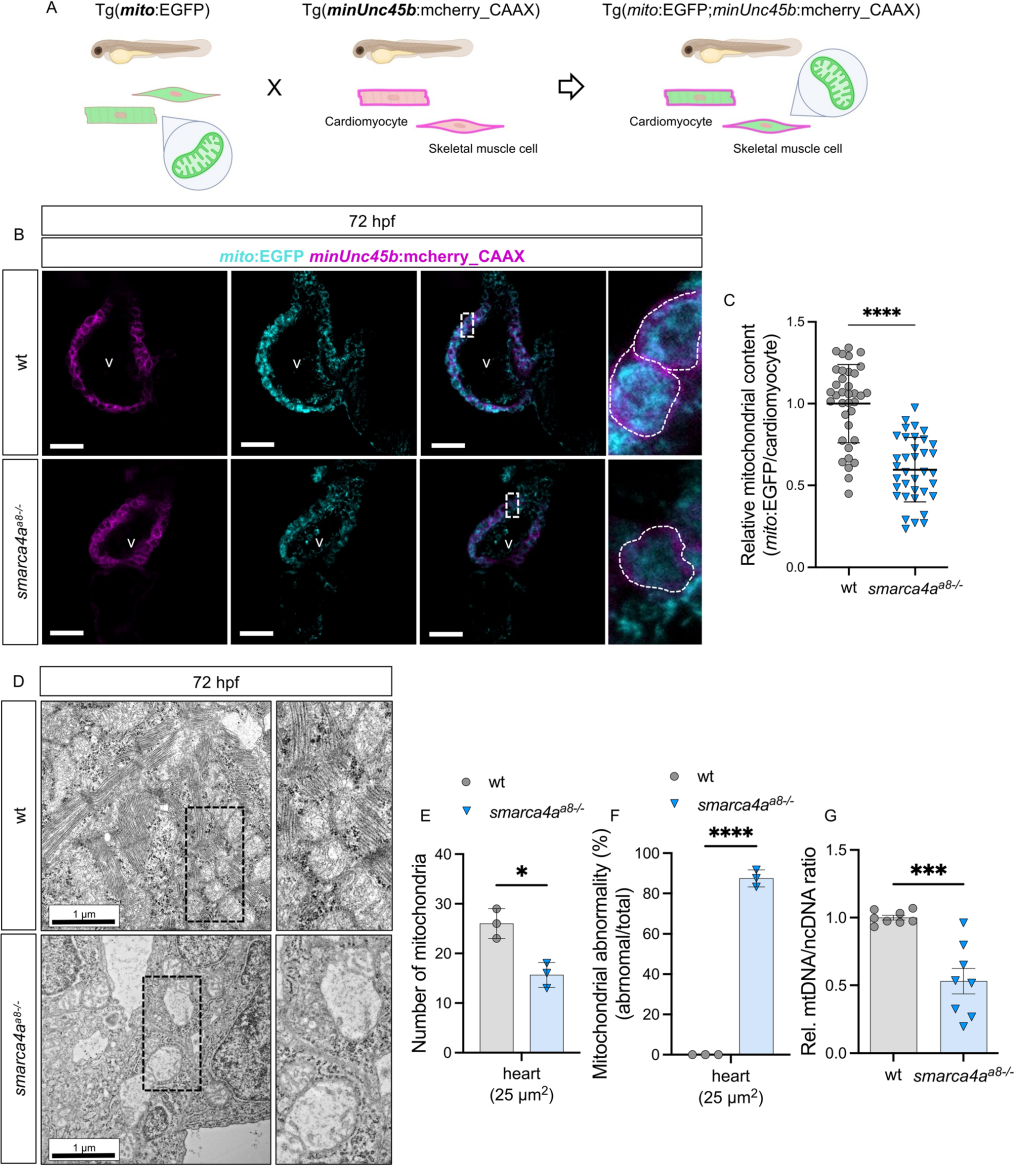
Mitochondria are impaired in the heart of *smarca4a*^*a8−/−*^ embryos. **A**. Schematic illustration of transgenic zebrafish lines used in subsequent experiments (Biorender.com). Enhanced green fluorescent protein (EGFP) is fused to a mitochondrial localization sequence (*mito*) to specifically label mitochondria, while mcherry is fused to myosin chaperone B (*minUnc45b*) and a CAAX motif to visualize the plasma membrane of myocytes. A transgenic zebrafish, Tg(*mito*:EGFP; *minUnc45b*:mcherry_CAAX) enables simultaneous imaging of mitochondrial distribution in myocytes. **B**. Confocal images of wt and *smarca4a*^*a8−/−*^ outcrossed with Tg(*mito*:EGFP; *minUnc45b*:mcherry_CAAX) at 72 hpf (scale bar: 50 μm). **C**. Quantitative analysis of mitochondrial content in an individual cardiomyocyte of wt and *smarca4a*^*a8−/−*^ (wt: 1.00 ± 0.24, *smarca4a*^*a8−/−*^: 0.60 ± 0.20, SD, *n* = 36). Mitochondrial distribution in cardiomyocytes is significantly decreased in *smarca4* mutants compared to wt siblings. **D**. Mitochondria in heart of wt and *smarca4a*^*a8−/−*^ visualized by means of electron microscopy at 72 hpf. **E**. Number of mitochondria in electron microscope images (D) of wt and *smarca4a*^*a8−/−*^ (wt: 26.00 ± 3.00, *smarca4a*^*a8−/−*^: 15.67 ± 2.52, SD, *n* = 3, *N* = 3, *p < 0.05). The number of mitochondria is decreased in heart tissue of *smarca4a*^*a8−/−*^ compared to wt. **F**. Ratio of abnormal mitochondria in heart of wt and *smarca4a*^*a8−/−*^ (wt: 0.05 ± 0.02, *smarca4a*^*a8−/−*^: 87.50 ± 4.17, SD, *n* = 3, *N* = 3, ****p < 0.0001). Abnormality of mitochondria in *smarca4a*^*a8−/−*^ is significantly increased compared to wt. **G**. Quantitative RT-PCR results showing decreased mitochondrial DNA/nuclear DNA (mtDNA/ncDNA) ratio in *smarca4a*^*a8−/−*^ compared to wt at 72 hpf (wt: 1.00 ± 0.05, *smarca4a*^*a8−/−*^: 0.53 ± 0.27, SD, *n* = 8, ***p < 0.001). Rel.=Relative

To investigate whether reduced transcriptional activity and diminished chromatin accessibility of mitochondria-related genes in *smarca4a*^*a8−/−*^ embryos lead to mitochondrial defects, we first assessed mitochondria distribution in heart and skeletal muscle of zebrafish embryos. To do so, we established a double transgenic zebrafish, Tg(*mito*:EGFP) [24] x Tg(*minUnc45b*:mcherry_CAAX), which allows visualization of mitochondria (EGFP fluorescence) and membrane of myocytes (mcherry fluorescence) (Fig. 4A). This double transgenic line was subsequently crossed with heterozygous *smarca4a*^*a8+/−*^ zebrafish, enabling the analysis of mitochondrial distribution in *smarca4a*^*a8−/−*^ embryos. We analyzed mitochondrial distribution in heart and skeletal muscle of *smarca4a*^*a8−/−*^ embryos at 72 hpf (Fig. 4B, S8A). Although *smarca4a*^*a8−/−*^ embryos did not show severe cellular deformation in cardiac and skeletal muscle tissues, mitochondrial fluorescence intensity was significantly reduced compared to wt siblings. Quantitative analysis of mitochondrial content in an individual cardiomyocyte or skeletal muscle cell further confirmed a significant reduction of mitochondria in *smarca4a*-deficent embryos (Fig. 4C, S8B).

To determine whether the decreased mitochondrial content resulted from structural disruption, we examined mitochondrial ultrastructure in the heart and skeletal muscle tissues using transmission electron microscopy (TEM). In both tissues, mitochondria in *smarca4a*^*a8−/−*^ embryos exhibited substantial structure damage, characterized by disrupted cristae architecture in comparison to wt embryos (Fig. 4D, S8C). Additionally, muscle fibers in *smarca4a* mutant embryos appeared disorganized, consistent with the IF staining results of Tropomyosin, F59, and F310 presented in Fig. S5E. Quantitative analysis of mitochondrial abundance in both striated muscle tissues demonstrated a significant reduction in *smarca4a*^*a8−/−*^ embryos compared to wt (Fig. 4E, S8D), with over 80% of mitochondria exhibiting abnormal structural features in heart as well as skeletal muscle tissues (Fig. 4F, S8E). To confirm decrease of mitochondria in *smarca4a*^*a8−/−*^ embryos, we performed qRT-PCR to analyze mitochondrial DNA (mtDNA) content relative to nuclear DNA (ncDNA). The mtDNA/ncDNA ratio was significantly reduced in *smarca4a*-deficient embryos compared to wt (Fig. 4G), indicating a lower mitochondrial abundance in *smarca4a*^*a8−/−*^ embryos.

**Fig. 5:**
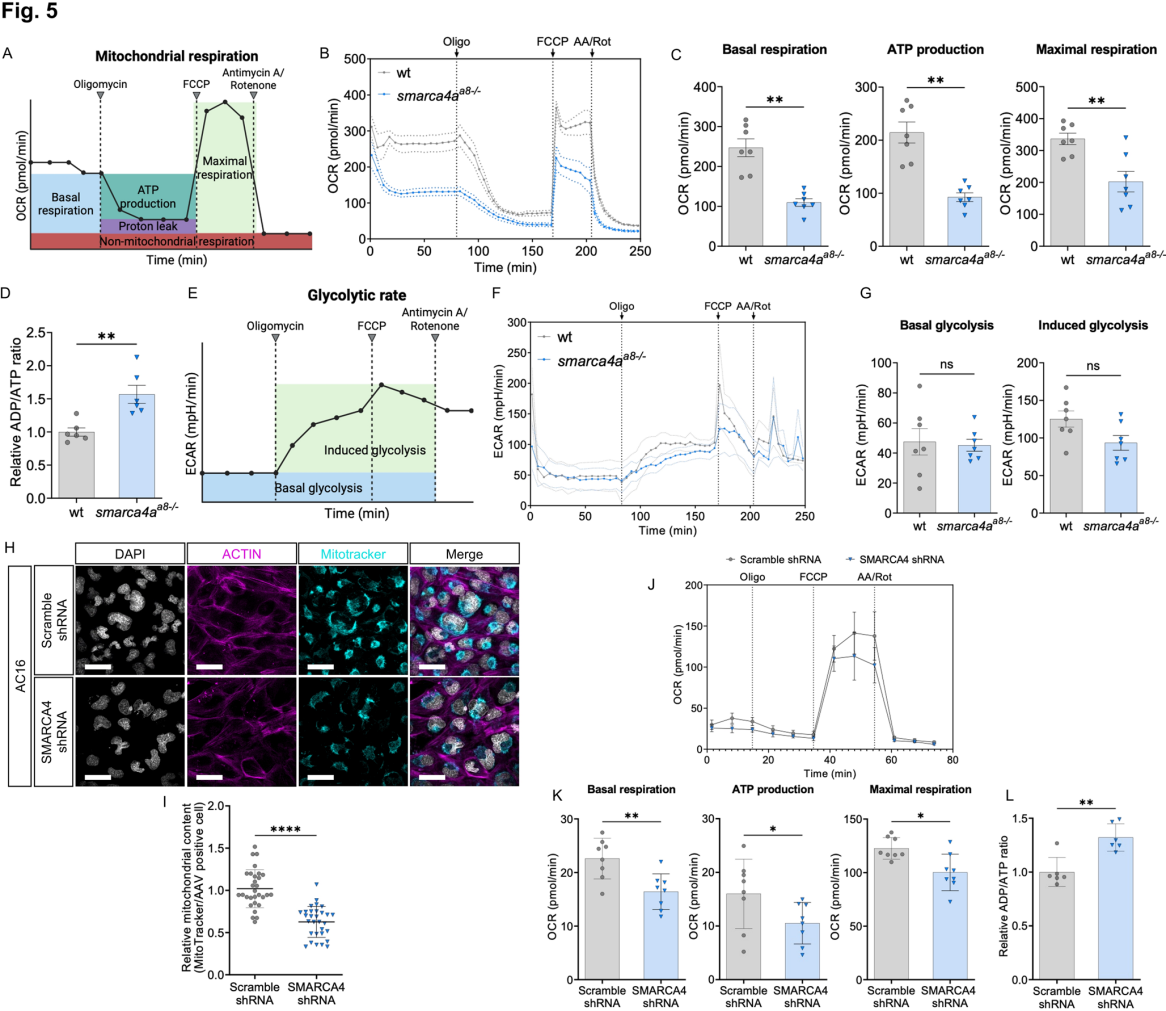
Mitochondrial respiration is reduced by loss of *smarca4*/*SMARCA4* in zebrafish embryos and human cardiomyocytes. **A** General depiction of mitochondrial stress assay (Seahorse XF Analyzer), showing the fundamental parameters of different mitochondrial respiratory modules (BioRender.com). OCR=oxygen consumption rate, FCCP=carbonyl cyanide p-trifluoro-methoxyphenyl hydrazone. **B** Seahorse XF mito stress test diagram of wt and *smarca4a*^*a8−/−*^ embryos at 72 hpf (SEM, *n* = 7). oligo=oligomycin, AA/Rot=Antimycin A/Rotenone. **C** Respective measurements of mitochondrial respiration. Basal respiration, ATP production, and maximal respiration are significantly reduced in *smarca4* mutants compared to wt embryos (Basal respiration: wt: 246.78 ± 58.31, *smarca4a*^*a8−/−*^: 109.62 ± 24.55; ATP production: wt: 214.39 ± 51.82, *smarca4a*^*a8−/−*^: 92.53 ± 21.88; Maximal respiration: wt: 336.30 ± 47.50, *smarca4a*^*a8−/−*^: 202.63 ± 84.77, SD, *n* = 7, **p < 0.01). **D** Relative ratio of ADP/ATP in wt and *smarca4* mutant embryos at 72 hpf (wt: 1.00 ± 0.15, *smarca4a*^*a8−/−*^: 1.57 ± 0.33, SD, *n* = 6, **p < 0.01). **E** Schematic description of parameters for analyzing glycolytic capacity from mito stress assay (BioRender.com). EACR=extracellular acidification rate. **F** Diagram showing ECAR measurement in wt and *smarca4a*^*a8−/−*^ embryos during mito stress test of (B). **G** Respective assessment of basal glycolysis and induced glycolysis in wt siblings and *smarca4a*^*a8−/−*^ embryos at 72 hpf (Basal glycolysis: wt: 47.39 ± 23.31, *smarca4a*^*a8−/−*^: 45.14 ± 10.42; Induced glycolysis: wt: 125.19 ± 28.42, *smarca4a*^*a8−/−*^: 93.39 ± 25.70, SD, *n* = 7, ns: *p* > 0.05). **H** Confocal images of human ventricular cardiomyocytes (AC16) at 72 h after transduction (hpt) of AAV-Scramble shRNA or -SMARCA4 shRNA. DAPI (grey), Actin (magenta), and MitoTracker (cyan) are visualized in fluorescence (scale bar: 50 μm). **I** Quantitative analysis of MitoTracker in AAV-Scramble shRNA or -SMARCA4 shRNA transduced human cardiomyocytes shows decreased mitochondrial content by SMARCA4 knockdown (Scramble shRNA: 1.02 ± 0.23, SMARCA4 shRNA: 0.63 ± 0.18, SD, *n* = 30, ****p < 0.0001). **J** Representative OCR graph of the seahorse mito stress assay using AAV-Scramble shRNA or -SMARCA4 shRNA transduced human cardiomyocytes (SEM, *n* = 7). **K** Analyzed OCR of AAV-Scramble shRNA or -SMARCA4 shRNA transduced cardiomyocytes showing basal respiration, ATP production, and maximal respiration (Basal respiration: Scramble shRNA: 22.59 ± 3.78, SMARCA4 shRNA: 16.41 ± 3.34; ATP production: Scramble shRNA: 15.99 ± 6.47, SMARCA4 shRNA: 10.52 ± 3.87; Maximal respiration: Scramble shRNA: 122.68 ± 10.06, SMARCA4 shRNA: 100.21 ± 17.01, SEM, *n* = 7, *p < 0.05, **p < 0.01). **L** Relative ratio of ADP/ATP in AAV-scramble shRNA or SMARCA4 shRNA transduced AC16 cardiomyocytes at 72 hpt (Scramble shRNA: 1.00 ± 0.14, SMARCA4 shRNA: 1.32 ± 0.13, SD, *n* = 6, **p < 0.01).

Next, we assessed whether loss of *smarca4a* leads to mitochondrial dysfunction in zebrafish embryos by performing a real-time mitochondrial respiration assay using a Seahorse XF96 pro Analyzer. For this, anesthetized zebrafish embryos were placed individually in each well of XF pro Analyzer cell culture microplate, and oxygen consumption rate (OCR) and extracellular acidification rate (ECAR) were measured under standardized time, inhibitor concentration, and temperature conditions [25]. The measured OCR from mitochondrial stress assay was analyzed by respective parameters (Fig. 5A-B). The results of the mitochondrial stress assay revealed significantly reduced basal respiration, ATP production, and maximal respiration in *smarca4a*-deficient embryos compared to wt siblings (Fig. 5C). Additionally, the relative ADP/ATP ratio was significantly increased in *smarca4a*^*a8−/−*^ embryos compared to wt (Fig. 5D), suggesting a deficit in ATP production. Given that ATAC-seq and RNA-seq analysis identified downregulated gene clusters associated with the *pyruvate metabolic process* (GO-term) and *glycolysis/gluconeogenesis* (KEGG pathway) in *smarca4a*^*a8−/−*^ embryos, we further analyzed ECAR measurements from the mitochondrial stress assay to evaluate glycolytic activity (Fig. 5E-F). However, quantitative analysis showed no significant differences in basal glycolysis or induced glycolysis between *smarca4a*-deficient embryos and wt siblings (Fig. 5G), indicating that the energy metabolic defect of *smarca4a*^*a8−/−*^ is because of mitochondrial dysfunction. Given that Smarca4/SMARCA4 has been implicated in protection against DNA damage induced by oxidative stress and in the regulation of redox homeostasis [26–28], we assessed reactive oxygen species (ROS) levels and apoptosis in *smarca4a*^*a8−/−*^ mutants. To this end, we performed DCFH-DA staining to quantify ROS levels and TUNEL assays to evaluate apoptotic cell death. Our results revealed a significant increase in ROS-related fluorescence intensity in *smarca4a*-deficient embryos compared to their wt siblings (Fig. S9), indicating elevated oxidative stress. However, TUNEL assays did not detect apoptotic cells in the *smarca4a*^*a8−/−*^ mutants (Fig. S10), suggesting that while *smarca4a* loss leads to increased oxidative stress, it does not induce apoptosis during zebrafish embryonic development.

Together, these findings demonstrate that *smarca4a*-deficient embryos exhibit impaired energy metabolism due to mitochondrial dysfunction, leading to deficits in ATP production and mitochondrial structural integrity.

### AAV-mediated knockdown of SMARCA4 causes mitochondrial defects in mammalian cells

To determine whether SMARCA4 also plays a crucial role in mitochondrial homeostasis and function in mammalian myocytes, we generated an adeno-associated virus (AAV)-based SMARCA4 shRNA vector to achieve stable SMARCA4 knockdown in human ventricular cardiomyocytes (AC16) and differentiated mouse skeletal muscle cells (C2C12) (Fig. S11). Following AAV-Scramble or AAV-SMARCA4 shRNA transduction, we performed IF staining using DAPI (nuclear marker), ACTIN (cytoskeletal marker), and MitoTracker (mitochondrial marker) to evaluate mitochondrial distribution in these cells. Interestingly, in both cardiomyocytes and differentiated skeletal muscle cells, mitochondrial content was significantly reduced in AAV-SMARCA4 knockdown cells compared to scramble shRNA controls (Fig. 5H-I, S12A-B). Next, we assessed mitochondrial respiration in AAV-transduced cells using a mitochondrial stress assay (Fig. 5J, S12C). In human cardiomyocytes, SMARCA4 knockdown led to a significant reduction in basal respiration, ATP production, and maximal respiration (Fig. 5K). By contrast, in differentiated skeletal muscle cells, only maximal respiration was significantly decreased (Fig. S12D). Despite these differences, ADP/ATP ratio assays revealed a significant defect in ATP production in both cell lines following SMARCA4 knockdown (Fig. 5L, S12E), indicating that loss of SMARCA4 impairs mitochondrial function, ultimately disrupting ATP production in mammalian myocytes.

## Discussion

In this study, we identified that the loss of *smarca4a* leads to developmental defects in both the heart and skeletal muscle of zebrafish, including pericardial edema, small heart chambers, and disorganized muscle fibers, which are indicative of cardiac and skeletal muscle myopathy. The absence of *smarca4a* resulted in suppressed chromatin accessibility and transcriptional activity of genes associated with mitochondrial homeostasis. Consequently, mitochondrial impairment was observed in the heart and skeletal muscle, leading to reduced mitochondrial respiration, not only in zebrafish embryos but also in mammalian myocytes.

SMARCA4, a core component of the Switch/sucrose nonfermentable (SWI/SNF) chromatin remodeling complex, is capable of remodeling nucleosome templates independently of other subunits [29]. It is a well-established key regulator of embryonic development, particularly in neuronal [5, 30–33] and cardiac [4, 6, 34] tissues. Other SWI/SNF complex subunits, such as PBRM1 and SMARCD3, and Smarce1, have also been implicated in cardiac development, particularly in chamber growth, maturation and heart morphogenesis [35–38]. While these studies highlight the role of Smarca4/SMARCA4 in development, its impact on energy metabolism during heart and skeletal muscle development remains unexplored. Notably, a recent study showed that SMARCA4 overexpression enhances glycolytic metabolism and the pluripotency of induced pluripotent stem cells [39]. These findings suggest that SMARCA4 may contribute to energy metabolism reprogramming.

Developmental processes such as differentiation, proliferation, and migration are highly dependent on ATP availability. During heart development, energy metabolism exhibits plasticity in response to environmental changes [40]. In fetal hearts, glycolysis predominates due to the limited availability of fatty acids and oxygen, whereas in neonatal and postnatal hearts, oxidative phosphorylation (OXPHOS) becomes the primary ATP-generating pathway [41]. This metabolic shift is accompanied by transcriptional changes, such as increased expression of hypoxia-inducible factor 1α (Hif-1α) and its downstream glycolytic enzymes [42], whereas fatty acid metabolism and mitochondrial biogenesis regulators, including PPARγ and PGC-1α, are upregulated in the postnatal heart [43]. Our bioinformatic analysis of developing zebrafish revealed reduced expression of genes involved in mitochondrial respiration, including *pparg*, *ppargc1a*, and *cox4i2*, consistent with our experimental evidence showing impaired mitochondrial respiration upon Smarca4 loss. Additionally, while glycolysis-related gene clusters (GO-term: *pyruvate metabolic process*, KEGG pathway: *glycolysis*) were also suppressed, the downregulation of key glycolytic regulators (*hif1a*, *hk1*, *pfk1*, and *ldha*) was not statistically significant, except for *slc2a1* (GLUT1) (data not shown). Moreover, ECAR analysis from the mitochondrial stress assay confirmed that glycolytic rates remained unchanged in *smarca4a*^*a8−/−*^ embryos. Interestingly, glycolysis inhibition does not significantly affect zebrafish embryonic development but rather impairs regenerative capacity [44], while mitochondrial respiration defects result in severe functional disorders [45, 46], suggesting that mitochondrial respiration is more contributing to development of zebrafish embryo than glycolysis.

As an ATP-dependent chromatin remodeler, SMARCA4 integrates metabolic cues into chromatin regulation. Energy metabolic disruptions caused by genetic mutations, mitochondrial stress, reactive oxygen species (ROS), or altered glucose metabolism influence SMARCA4 expression, thereby facilitating SWI/SNF complex recruitment to active enhancers and transcriptional start sites (TSS), ultimately enhancing gene transcription [47–50]. In tumor cells, SMARCA4 has been identified as a regulator of mitochondrial metabolism [51]. SMARCA4-deficient tumor cells exhibit dysregulation of key OXPHOS genes (e.g., PGC-1α, ATP5L) and oxidative stress response genes (e.g., GSTO7, GSTO1), leading to compromised mitochondrial respiration [52]. Additionally, SMARCA4 deletion upregulates genes associated with mitochondrial degradation, thereby disrupting redox homeostasis [53]. Bultmann et al. further demonstrated that SMARCA4 maintains cardiomyocyte homeostasis by regulating mitochondrial dynamics and mitophagy. In SMARCA4/SMARCA2 double-knockout mouse hearts, mitochondria were fragmented, reduced in number and size [54]. While these findings suggest an essential role for SMARCA4 in mitochondrial biogenesis, direct mechanistic insights linking SMARCA4 to mitochondrial function remain limited. Here, we provide experimental evidence that Smarca4 loss suppresses chromatin accessibility and transcriptional activity of genes involved in mitochondrial respiration (*ppargc1a*, *pparg*, *cox4i2*), mitochondrial dynamics (*mfn1a*, *mfn2*), and mitochondrial DNA regulation (*nrf1*). Motif enrichment analysis of downregulated differentially accessible regions (DARs) further revealed significantly enriched transcription factor binding sites for NRF1 and USF family members - both of which are established regulators of mitochondrial biogenesis and oxidative phosphorylation. NRF1 directly activates nuclear - encoded mitochondrial genes, including those encoding respiratory chain subunits and components of the mitochondrial transcriptional machinery, and its functional depletion leads to impaired mitochondrial morphology and bioenergetics [16, 17, 55]. USF factors, particularly USF2, have been shown to transcriptionally regulate the ATP synthase α-subunit gene [14] and to maintain mitochondrial function in cardiomyocytes [15]. Notably, USF and NRF1 have been reported to interact at shared promoter sites, including the FMR1 promoter, in a methylation-dependent manner, suggesting possible cooperative regulation of gene expression [15, 56]. The concurrent reduction in chromatin accessibility at these motifs and downregulation of their target genes suggests that loss of Smarca4/SMARCA4 disrupts key transcriptional networks governing mitochondrial homeostasis. We further demonstrate that Smarca4/SMARCA4 depletion impairs mitochondrial function and respiration in both in vivo and in vitro models, underscoring a direct mechanistic link between Smarca4/SMARCA4, transcription factor-mediated regulation of mitochondrial genes, and maintenance of mitochondrial integrity. Although our data reveal a strong association between Smarca4 loss, reduced chromatin accessibility at mitochondrial gene loci, and impaired mitochondrial function, we cannot completely exclude that some of the observed mitochondrial phenotypes may arise secondarily from broader developmental defects. In the zebrafish model, analyses were performed at 72 hpf, a stage at which Smarca4-deficient embryos already exhibit cardiac and skeletal muscle abnormalities. Thus, reduced chromatin accessibility and transcriptional repression of mitochondrial genes may reflect either a direct consequence of Smarca4 loss or downstream effects associated with compromised cellular homeostasis. In addition, as chromatin accessibility analyses were performed exclusively on isolated hearts, the present data do not allow conclusions regarding ubiquitous or pleiotropic Smarca4-dependent regulation of mitochondrial genes across other tissues.

In mammals, SMARCA2 is utilized as an alternative of SMARCA4 to remodel targeted chromatin [50]. SMARCA4 and SMARCA2 share 75% amino acid sequence homology [57], and SMARCA2 can compensate for SMARCA4 loss in certain cellular contexts [58]. However, during embryonic development, SMARCA4 appears indispensable, as SMARCA2 cannot fully substitute for its function [5, 59, 60]. Our findings suggest that while SMARCA4 is essential for cardiac and skeletal muscle development, SMARCA2 may compensate for mitochondrial homeostasis in mammalian cells. Indeed, AAV-mediated SMARCA4 knockdown reduced mitochondrial distribution, respiration, and ATP production in AC16 and C2C12 cells, albeit less severely than in zebrafish embryos. Notably, Western blot analysis revealed increased SMARCA2 expression following SMARCA4 knockdown, supporting a potential compensatory role of SMARCA2 in mitochondrial homeostasis.

These findings highlight a previously unrecognized role of Smarca4/SMARCA4 in mitochondrial function and energy metabolism, with implications for muscle development and potentially also cardiac regeneration.

## Experimental Model and Subject Details

### Animals

All procedures and experiments in this study were carried out after appropriate institutional approvals (Tierforschungszentrum (TFZ) Ulm University; No. 0183, 24.03.2011; Regierungspräsidium Tübingen; No. 1415) and national (Germany) ethical and animal welfare regulation (Tierschutzgesetz § 11). The experimental procedures in this study were performed according to the guidelines from the EU Directive 2010/63/EU on the protection of animals used for scientific purposes. Care and breeding of zebrafish Danio rerio were carried out as described [61]. For injection experiments the TüAB wild-type strain was used. Adult *smarca4a*^*a8−/−*^ mutants were kept as heterozygous fish, breeding resulted in 25% homozygous *smarca4a* mutant offspring. Pictures or movies were recorded at 48, 72 and 96 h post fertilization (hpf). For documentation, zebrafish embryos were treated with 0.003% 1-phenyl-2-thiourea to inhibit pigmentation. For immunofluorescence (IF) or heart-specific analyses, the Tg(*mito*:EGFP), Tg(*minunc45b*:GFP) or Tg(*myl7*:mcherry.nls;*fli1*:EGFP) line were used, marking cardiomyocytes with mcherry or GFP expression.

### Cell culture

AC16 cells (human ventricular cardiomyocyte cell line) were cultured in DMEM/F12 GlutaMax, supplemented with 12.5% fetal calf serum (FCS), 1% penicillin/streptomycin and incubated in a condition of 5% CO_2_, 95% humidity at 37 °C. The medium was refreshed every 48 h and cells were maintained as subcultures using 0.05% trypsin/0.02% EDTA when they reached confluence. AC16 cells were subsequently maintained until passage 10th for research to avoid senescence.

C2C12 cells (murine skeletal myoblasts) were cultured in DMEM, supplemented with 20% FCS, 1% penicillin/streptomycin and incubated in a condition of 5% CO_2_, 95% humidity at 37 °C. C2C12 cells were differentiated in low-serum (2% FCS) differentiation medium. Differentiation medium was refreshed every 24 h and the C2C12 cells were used for experiments after 5 days of differentiation.

### Method details

#### Heart rate and fractional shortening

We measured fractional shortening by video microscopy at RT and Image J. The diameters (short axis and long axis) of the ventricular lumen at the end of contraction (systole) and relaxation (diastole) were compared as described before [46].

### Microinjection

Microinjections were performed into fertilized zebrafish oocytes at the 1-cell stage, using pulled glass capillaries (World Precision Instruments) and a Microinjector (Eppendorf). Embryos were then allowed to develop at 28.5°C until the indicated stages. To inhibit pigmentation, 0.003% 1-phenyl-2-thiourea was added to the regular embryo medium E3 (5 mM NaCl, 0.17 mM KCl, 0.33 mM CaCl2, 0.33 mM MgSO4 dissolved in water). Morpholino-modified antisense oligonucleotides (MOs; Gene Tools, LLC) were injected into one-cell stage zebrafish embryos. To knock-down zebrafish *Smarca4a*, MO targeting the translational start site (*smarca4a*-MO: 5´-TCAGGAGTGGACATCTCTCAGCAGA-3´) or standard control MO (control-MO: 5´-CCTCTTACCTCAGTTACAATTTATA-3’) were injected into fertilized oocytes at the one-cell stage. MOs were injected with 2.5 ng in 0.2 M potassium chloride.

### Compound treatment

PFI3, bromodomain inhibitor, was dissolved in DMSO at concentration of 100 mM as stock solution. DMSO or 100 µM of PFI 3 were diluted in E3 media (5 mM NaCl, 0.17 mM KCl, 0.33 mM CaCl_2_, 0.33 mM MgSO_4_) and treated to embryos from 0 hpf to 72 hpf. The E3 media containing DMSO or PFI3 was replaced every 24 h.

### Ca^2+^ transient analysis

Wild-type siblings and *smarca4a*-mutants were anesthetized with tricaine and mounted in 2% low melting point agarose ventral-side down on glass coverslips. Heart contraction was arrested by incubating the mounted embryos in 50 µM blebbistatin for 2 h prior to imaging [62]. Measured calcium currents were analyzed using ImageJ and Clampfit10.7 software. The results were visualized by GraphPad.

### Protein lysate extraction and Western blot analysis

For each of independent Western blot experiments, 50 embryos at 72 hpf or 100 embryonic hearts from wildtype (wt) siblings and *smarca4a*^*a8−/−*^ mutant zebrafish, were used, respectively. Whole embryos were deyolked and washed before homogenized in RIPA buffer containing protease inhibitor and phosphatase inhibitor. After incubation on ice for 30 min, samples were centrifuged for 15 min at 18,000 *g* and 4 °C. Supernatant was collected and measured by Bradford assay. AC16 cells and differentiated C2C12 cells were cultured in 6-well plates. Washed cells with PBS were dissociated in cold PBS using cell scrappers and collected in 1.5 ml tubes. Harvested cells were centrifuged at 800 *g* and 4 °C and resuspended with RIPA as described above. For Western blot analysis protein lysates were boiled in 5× (protein lysate of whole mount embryos or AC16/C2C12 cells) or 2× (embryonic heart tissues) Laemmli Buffer and loaded on a precast 8–16% SDS gel. The membrane was blocked in 5% skim milk powder in TBST (TBS with 0.1% Triton) for 2 h at room temperature (RT). Primary antibodies were incubated over night at 4 °C. The corresponding anti-rabbit or -mouse IgG HRP-linked secondary antibodies were incubated for 2 h at RT. Signals were detected by a luminescent image analyzer. Western blot bands were quantified by using ImageQuant LAS4000 software and normalized to β-Actin or Vinculin of each protein targets and controls (wt or Scramble shRNA).

### Histology

For histology, embryos were fixed in 4% PFA (in PBS), embedded in JB-4 and 5 μm sections were cut using Leica RM2255 microtome, dried and stained with hematoxylin & eosin. The sections were visualized by Axioskop 2 plus.

### Immunofluorescence (IF) staining

To perform IF staining, embryos were prepared at 72 hpf and fixed in 4% PFA overnight at 4 °C. Afterwards, samples were permeabilized in 0.1% Triton in PBS (PBT) and blocked in 5% normal goat serum (NGS) in PBT. Anti-MF20 (1:50), -S46 (1:200), -tropomyosin (1:200), -F59 (1:50), or -F310 (1:50) primary antibody was attached, followed by corresponding Alexa-488 labelled secondary antibody. Hearts were dissected and mounted on the slide with VectorShield while whole embryo were mounted on 2% low-melting agarose for confocal imaging. For MitoTracker assay, AC16 and differentiated C2C12 cells were incubated with 120 nM of MitoTracker™ Deep Red in culture media for 1 h before fixation with 4% PFA overnight at 4 °C. Cells were permeabilized in PBT and blocked in 3% BSA in PBT. SIR-Actin (1:1000) and Hoechst (1:2000) were applied before visualization using confocal.

### Touch response assay

The motility of embryos was assessed at 72 hpf. Each embryo was placed in the center of a petri dish filled with E3 media on a thermoplate (at 28 °C). The embryo’s reaction stimulated by touch using pipet-tip was recorded until the embryo stops movement. To assess the average swim speed and distance travelled, the recorded video was analyzed with the Tracker software.

### RNA extraction and quantitative real-time PCR

Per biological replicate, a pool of 25 embryos and 100 embryonic hearts were collected at 72 hpf. RNA extraction was carried out by RNeasy Mini Kit according to the manufacturer’s instructions. Total RNA (200 ng) was reverse transcribed to produce cDNA using Superscript III reverse transcriptase. Quantitative real-time PCR was carried out according to standard protocols using SYBR Green on a LightCycler 480 II (Roche). House-keeping genes, β-actin or 18s ribosomal RNA, were used as reference gene for normalization of gene expression (List of primer sequences; Supplementary Table 2).

Mitochondria were quantified by comparing the copy number of the nuclear gene (ncDNA) and the representative mitochondrial gene (mtDNA). Therefore, isolated genomic DNA was used as input and quantitative real-time PCR was performed according to the standard protocol using SYBR Green on a LightCycler 480 II.

### Library preparation and sequencing

For RNA-seq, three independent RNA samples for *smarca4a* mutants and siblings were isolated and the RNA was extracted. The libraries for RNA-seq were generated and sequenced by the Eurofins.

#### Bioinformatics analysis of RNA-seq

Processing of raw FASTQ files and alignment to Ensembl GRCz10 was done by the Genomics Core Facility at University Ulm. After creating the count matrix for Ensembl IDs and samples, low count reads (< ten counts per million in less than 3 samples) were removed. Counts were TMM normalized using the R/Bioconductor package edgeR [63]. The count data were further processed using the voom transformation, lmfit and eBayes functions included in the R/Bioconductor package limma [64]. The calculated DEGs were annotated using the R/Bioconductor package AnnotationDbi [65]. For comparison of wt and *samrca4a*^*a8−/−*^ samples the mean value of the counts in each group were color coded for selected genes in a heatmap. The number of up- and down-regulated DEGs were counted, filtering for fold change (|log2(fold change)| > 1) and false discovery rate (FDR) < 0.05 and displayed in a bar plot. To test for suppression of gene ontology (GO) terms, the goana function of the limma package was used. GO terms and KEGG pathways were shown in enrichment maps according to the p-values and gene counts.

### ATAC sequencing procedure and data analysis

For ATAC sequencing, 100 hearts of *smarca4a*^*a8−/−*^ mutants and siblings were dissected and flash frozen to send to Active Motif for ATAC-seq library preparation and sequencing. Adapters and low‑quality bases were removed, mitochondrial/alignment artifacts filtered, and duplicates marked. Peaks were called per sample and merged across conditions to a consensus peak set. Peak accessibility was quantified as Tn5 insert pileups per consensus peak and analyzed with a quasi‑likelihood negative binomial model. Differentially accessible regions (DARs) were defined at FDR < 0.05 with an absolute logFC threshold; for inactivation‑focused analyses we emphasized decreased accessibility using logFC ≤ − 1. Peaks were annotated relative to the nearest transcription start site (TSS). Promoters were defined as TSS ± 2 kb; distal elements were peaks outside this window. When needed, gene–peak links were assigned by nearest TSS and, for distal peaks, constrained to the same topologically associated domain if available. GRCz11 (NCBI) was used as reference for further analyses. For differential analysis (e.g. heatmap), maxtag ((average value×length of region)/224, cutoffs: 100–200) and the position were considered for comparison. Average value of the tags per sample were compared and visualized using R. Chromatin regions that are differentially open in specific regions of interest were visualized and identified using Integrative Genomic Viewer.

#### DEG–DAR integration and Motif enrichment with AME

We intersected DARs with the set of DEGs. For promoter analyses, only DARs overlapping TSS ± 2 kb of DEGs were retained. This yielded the integrative peak list used for motif discovery.

Motif enrichment was performed with AME (MEME Suite v5.5.8) on foreground FASTA sequences against curated vertebrate TF motif libraries. The primary database was JASPAR 2024 CORE vertebrates, non‑redundant (v2); auxiliary analyses optionally included Jolma 2013 and UniPROBE mouse motif sets to assess robustness. Enrichment scoring was based on the total number of motif hits per sequence exceeding an internally determined threshold (“total-hits” method). Statistical significance was evaluated using Fisher’s exact test, comparing motif hit frequencies in the foreground sequences to those in a shuffled background. The hit threshold was optimized by AME’s internal true-positive (TP) threshold estimation (–hit-lo-fraction 0.25), which selects a position weight matrix (PWM) score cutoff that maximizes discriminatory power while minimizing low-score hits. Multiple testing correction was performed using the Benjamini–Hochberg false discovery rate (FDR) method, and motifs with an adjusted P-value (adj. P) < 0.05 were considered significantly enriched. For each motif, we also report the nominal P-value, E-value, TP (%) and FP (%) - representing the fraction of foreground and background sequences, respectively, containing at least one above-threshold motif occurrence - to facilitate interpretation.

#### Single cell RNA sequencing and data analysis (embryonic hearts)

For heart dissection, wt and *samrca4a* deficient embryos were collect and anesthetized with tricaine at 72 h. By use of 1 ml syringe with 30G 1/2” (13 mm) needle, 150–200 hearts were manually dissected from wt and *smarca4a* mutant embryos. Dissected hearts were collected in 1.5 ml tube containing cold DMEM and centrifuged at 700 rcf, 4°C, for 5 minutes. Supernatant was removed and cold HBSS was added for washing, then the samples were centrifuged again with same condition. After removing supernatant, the dissected hearts were incubated with mixed enzymes (100 µl of papain & 5 µl of thermolysin) in a Thermomixer at 300 rpm, 37°C, for 40 minutes to dissociate cells. Supplemented DMEM (+ 10% FBS) was used to quench the reaction and centrifuged at 700 rcf, 4°C, for 5 minutes. After aspiration of supernatant, the dissociated cells were suspended with PBS (+ 0.04% BSA) and counted. For single-cell based mRNA extraction, cDNA amplification, and library preparation we used SCOPE-chip (SD) RNA library kit by following manufacturers’ protocol. Dissociated cells, concentrated 2.0–3.0 ⋅ 10^5^ cells/ml in average, were dispensed into microwell chip to capture 7,000–9,000 cells.

To filter the data, we used following filtering options: expressed gene range: mingene = 200, maxgene = 98% genes of each sample; expressed UMI range: minUMI = 0,maxUMI = 98% UMI of each sample; mitochondrial content: the threshold (5, 10, 15, 20, 30, 50), that is closest to filtering 5% of the cells. To normalize gene expression for data integration, we removed ribosomal protein genes (RPL and RPS) and used the standard Seurat processing guidelines [66]. Integrated data sets were annotated with referenced cell type markers [67], and visualized by UMAP. Further analyses including gene expression analysis, DEGs analysis, and GO-term analysis were visualized by CeleLens Could (Singleron).

#### Transmission electron microscopy

For transmission electron microscopy analyses, zebrafish embryos were fixed in 2.5% glutaraldehyde and 1% paraformaldehyde in 0.1 M phosphate buffer (pH 7.3). Afterwards, samples were post-fixed in 1% osmium tetroxide. Embryos were embedded in Epon and then sections and ultrathin Sect. (50 nm) were cut. The ultrathin sections were contrasted using uranyl acetate and lead citrate and subsequently imaged on an electron microscope JEM-1400 (JEOL USA, Inc., Peabody, MA, USA) [68].

### Seahorse XF96 assay

Embryos were incubated in 125 mg/ml tricaine containing E3 media before transferred to Seahorse XF96 cell culture plate with a 175 µl of XF base medium with minimal DMEM to each well. The central position of the embryos was checked. The temperature of the analyzer was maintained at 28 °C. Measurement protocol was followed by standardized conditions [25].

### Reactive oxygen species measurement

ROS level was assessed by staining wt and *smarca4a*^*a8−/−*^ embryos with 10 µM of the fluorescent dye 2,7-dichloro-dihydro-fluorescein-diacetate (DCFH-DA) green-fluorescent dye in the dark for 30 min. After incubation, embryos were washed three times with E3 medium and anesthetized with tricaine. The green fluorescence of oxidized DCF of embryos was imaged and quantified by ImageJ.

## TUNEL assay

TUNEL assays were performed using DeadEnd Fluoro metric TUNEL System on dissected zebrafish hearts. Positive control was additionally incubated with DNases and negative control was proceeded without rTdT enzyme incubation.

### Statistical analysis

All graphs and statistical analyses are expressed as means ± standard derivation (SD) or standard error of means (SEM). Analyses were performed using one-way ANOVA or two-way ANOVA. When necessary, data were expressed as means ± SD or SEM of at least three independent experiments, and statistical analysis for single comparison was performed using the Student’s t-test (Mann-Whitney test). Statistical significance was determined by the Holm-Sidak method, a value of *p* < 0.05 was accepted as statistically significant.

## Supplementary Information

Below is the link to the electronic supplementary material.

Supplementary Material 1 Supplementary Table S1. Key resources table. List of primer sequences

Supplementary Material 2 Figure. S1: Mutation of smarca4 leads to cardiac and skeletal muscle phenotype during early development of zebrafish embryos. Figure. S2: Loss of Smarca4 decreases cardiomyocyte proliferation in developing heart of zebrafish.

Supplementary Material 3 Figure. S3: Morpholino-mediated Smarca4 inhibition recapitulates cardiac defects in zebrafish embryos. Figure. S4: Loss of smarca4 reduces calcium flux in zebrafish embryonic heart.

Supplementary Material 4 Figure. S5: Ablated Smarca4 impairs skeletal muscle development and motility in zebrafish embryo.

Supplementary Material 5 Figure. S6: Inhibitory effect of smarca4a MO or PFI3 impairs skeletal muscle development and motility in zebrafish embryo.

Supplementary Material 6 Figure. S7: Heart-specific RNA-seq reveals suppressed gene ontology (GO) terms of muscle contraction and ATP production in smarca4aa8-/- hearts.

Supplementary Material 7 Figure. S8: Mitochondria are impaired in skeletal muscle of smarca4aa8-/-.

Supplementary Material 8 Figure. S9: Reactive oxygen species (ROS) level is increased in smarca4aa8-/-. Figure. S10: Smarca4 mutation does not affect DNA damage induced apoptosis in zebrafish embryonic heart.

Supplementary Material 9 Figure. S11: Efficient knockdown of SMARCA4 by AAV-SMARCA4 shRNA transduction in mammalian cardiomyocytes and skeletal muscle cells. Figure. S12: AAV-mediated SMARCA4 knockdown decreases mitochondrial respiration in mammalian skeletal muscle cells.

## Data Availability

All supporting data are available within the article and its Supplemental Material. Detailed materials and methods can be found in the Supplemental Material. ATAC-seq and RNA-seq data from this study have been deposited in the Gene Expression Omnibus database under accession number GSE223722. Resource Availability: Further information and requests for resources and reagents should be directed to and will be fulfilled by the Lead Contact, Steffen Just (steffen.just@uniklinik-ulm.de). Material Availability: All unique/stable reagents generated in this study are available from the Lead Contact with a completed Materials Transfer Agreement (Key resources table; Supplementary Table S1).
