# Supplementary material for: Smarca4 maintains mitochondrial homeostasis and energy metabolism during cardiac development
Source: Cell Mol Life Sci. 2026 Mar 7;83(1):167. doi: 10.1007/s00018-026-06168-3 (PMC13013801; doi:10.1007/s00018-026-06168-3)
Supplement: Supplementary file 1 — Supplementary Material 1 Supplementary Table S1. Key resources table. List of primer sequences [file 18_2026_6168_MOESM1_ESM.docx]

**Supplementary Table S1.** Key resources table

| **REAGENT or RESOURCE** | **SOURCE** | **IDENTIFIER** |
| --- | --- | --- |
| **Chemicals** | | |
| Proteinase K, recombinant, PCR Grade | Roche | Cat#3115844001 |
| 1-Phenyl-2-thiourea | Sigma-Aldrich | Cat#P7629 |
| Ethyl-3-aminobenzoate methanesulfonate salt | Sigma-Aldrich | Cat#A5040 |
| Paraformaldehyde (PFA) | Sigma-Aldrich | Cat#158127 |
| JB-4® Embedding Kit | Polyscience | Cat#00226-1 |
| Methyl Cellulose | Sigma-Aldrich | Cat#64632 |
| Trypsin (powder) | Biochrom | Cat#L2103 |
| VECTASHIELD® HardSet™ with DAPI | Vector Laboratories | Cat#H-1500 |
| Normal Goat Serum (NGS) | Sigma-Aldrich | Cat#S26-100ML |
| Triton^®^ X-100 | Serva Electrophoresis | Cat#37240 |
| Invitrogen^TM^ RNaseOUT™ Recombinant Ribonuclease Inhibitor | Thermo Fisher Scientific | Cat#10777-019 |
| Light Cycler 480 SYBR Green I Master Mix | Roche | Cyt#04887352001 |
| Nuclease free water | QIAGEN | Cat#129114 |
| cOmplete™, EDTA-free Protease Inhibitor | Roche | Cat#04693132001 |
| PhosSTOP™, Phosphatase Inhibitor | Roche | Cat#04906837001 |
| RIPA buffer (10x) | Cell Signaling | Cat#9806S |
| Protein Assay Dye Reagent Concentrate (Bradford) | Bio-Rad | Cat#5000006 |
| 8–16% Mini-PROTEAN® TGX™ Precast Protein Gels, 10-well | Bio-Rad | Cat#456-1104 |
| 10x Tris/Glycine/SDS | Bio-Rad | Cat# 1610732 |
| 10x Tris/Glycine Buffer | Bio-Rad | Cat# 1610734 |
| PageRuler™ Plus Prestained Protein Ladder | Thermo Fisher Scientific | Cat# 26619 |
| TWEEN 20 | Sigma-Aldrich | Cat#P1379 |
| Skim Milk Powder | Sigma-Aldrich | Cat#70166 |
| Amersham™ ECL Prime | Cytiva | Cat#RPN2232 |
| Immobilon-P PVDF-Membran | Millipore | Cat#IPVH00010 |
| Fibronectin from bovine plasma | Sigma Aldrich | Cat#F1141 |
| Gibco^TM^ PBS | Thermo Fisher Scientific | Cat#14190-094 |
| Gibco^TM^ DMEM/F12 1:1 GlutaMax | Thermo Fisher Scientific | Cat#31331-028 |
| Gibco^TM^ DMEM | Thermo Fisher Scientific | Cat#10829-018 |
| FBS SUPERIOR stabil^®^ | Biochrom | Cat#S0615 |
| Gibco^TM^ Penicillin-Streptomycin | Thermo Fisher Scientific | Cat#15140122 |
| Trypsin 0.05 %/EDTA 0.02 % in PBS (without Ca, Mg) | PAN Biotech | Cat#P10-023100 |
| Collagenase A | Roche | Cat#10103586001 |
| Pancreatin | Sigma-Aldrich | Cat#P1750 |
| PFI3 | Selleckchem | Cat#S7315 |
| blebbistatin | Abcam | Cat#ab120425 |
| DCFH-DA | Sigma-Aldrich | Cat#35845 |
| Low-melting agarose | Promega | Cat#V2111 |
| Primary cardiomyocyte isolation kit | Thermo Fisher Scientific | Cat#88281 |
| **Antibodies** | | |
| BRG1 | Signalway Antibody | Cat#49101 |
| Anti-beta-actin | Sigma Aldrich | Cat#A5441 |
| BRG1/SMARCA4 (D1Q7F) | Cell Signaling | Cat#49360 |
| BRM/SMARCA2 (D9E8B) | Cell Signaling | Cat#11966 |
| Vinculin | Abcam | Cat#Ab91459 |
| Anti-mouse IgG, HRP-linked | Cell Signaling | Cat#7076S |
| Anti-rabbit IgG, HRP-linked | Cell Signaling | Cat#7074S |
| Tropomyosin (CH-1) | Hybridoma bank | Cat#CH-1 concentrate |
| F59 (myosin heavy chain) | Hybridoma bank | Cat#F59 concentrate |
| F310 (myosin light chain) | Hybridoma bank | Cat#F310 concentrate |
| Goat anti-Mouse IgG1 Cross-Adsorbed Secondary Antibody, Alexa Fluor 488 | Thermo Fisher Scientific | Cat#A-21121 |
| Goat anti-Mouse IgM (Heavy chain) Cross-Adsorbed Secondary Antibody, Alexa Fluor 555 | Thermo Fisher Scientific | Cat#A-21426 |
| Goat anti-Rabbit IgG (H+L) Highly Cross-Adsorbed Secondary Antibody, Alexa Fluor 488 | Thermo Fisher Scientific | Cat#A-11034 |
| Goat anti-Rabbit IgG (H+L) Highly Cross-Adsorbed Secondary Antibody, Alexa Fluor 555 | Thermo Fisher Scientific | Cat#A-21429 |
| SiR-actin kit | Spirochrome | Cat#SC001 |
| MitoTracker™ Deep Red FM | ThermoFisher | Cat#M22426 |
| Hoechst 33342 | ThermoFisher | Cat#H3570 |
| **Critical Commercial Assays** | | |
| Morpholino | GENE TOOLS, LLC | https://www.gene-tools.com |
| RNeasy Micro Kit | QIAGEN | Cat#74004 |
| Superscript III Reverse Transcriptase | Thermo Fisher Scientific | Cat#18080-085 |
| Isolate II PCR and Gel Kit | Biocat | Cat#BIO-52060 |
| ATAC-seq service | Active Motif | https://www.activemotif.com |
| scRNA-seq service | Singleron | https://singleron.bio |
| TubeSeq service | Eurofins Genomics | https://eurofinsgenomics.eu/ |
| Next Generation Sequencing (RNA sequencing) | Genomics Core Facility at University Ulm | https://www.uni-ulm.de/medizinische-fakultaet/forschung/core-facilities/genomics/ |
| DeadEnd Fluoro metric TUNEL System | Promega | Cat#G3250 |
| **Experimental Models: Animal** | | |
| Zebrafish | Gregg et al.(10) | *smarca4a^a8-/-^* |
|  | Kim et al.(24) | Tg(*mito*:EGFP) |
|  | Steffen Just lab (this paper) | Tg(*minUnc45b*:EGFP.CAAX) |
| **Experimental Models: Cell line** | | |
| AC16 | Sigma Aldrich | Cat#SCC109 |
| C2C12 | ATCC | Cat#CRL-1772 |
| **Oligonucleotides** | | |
| Oligo dTs | Eurofins | N/A |
| dNTP Mix | Thermo Fisher Scientific | Cat#R1122 |
| **Software and Algorithms** | | |
| R (version 3.5.1) | CRAN | https://www.r-project.org/ |
| edgeR | R/Bioconductor | https://rdocumentation.org/packages/edgeR/versions/3.14.0 |
| limma | R/Bioconductor | https://rdocumentation.org/packages/limma/versions/3.28.14 |
| AnnotationDbi | R/Bioconductor | https://rdocumentation.org/packages/AnnotationDbi/versions/1.34.4 |
| Integrative Genomic Viewer | Broad Institute  and the Regents of the  University of California | http://software.broadinstitute.org/software/igv |
| GraphPad Prism (version 9) | GraphPad Software, La Jolla California, USA | https://www.graphpad.com:443 |
| ImageJ | NIH (61) | https://imagej.nih.gov/ij/ |
| pCLAMP^TM^10.7 | Molecular Devices | https://www.moleculardevices.com/products/axon-patch-clamp-system/acquisition-and-analysis-software/pclamp-software-suite |
| Tracker | Open-Source-Physics (OSP) Java framework | https://opensourcephysics.github.io/tracker-website/ |
| **Adeno-Associated Viruses** | | |
| AAV6-Scramble shRNA | VectorBuilder | https://en.vectorbuilder.com/ |
| AAV6-hSMARCA4 shRNA |  |  |

**Supplementary Table S2.** List of primer sequences

| **Experiment** | **Gene** | **Forward sequence (5´-3´)** | **Reverse sequence (5´-3´)** |
| --- | --- | --- | --- |
| Zebrafish | *smarca4a* | GGCCCTCAAAGACAAGAAGA | CATTTGACTAGTCTCATCCAGAGG |
|  | *cox4i2* | GGTCGGAGACGCTAGAATGT | AGTAGTCCTCGACCTTCGCA |
|  | *pparg* | AGCTACAGCCCTGAGGAGAA | GAGGAGATTCGGGCTCAAG |
|  | *ppargc1a* | CCTGCTAACTCCCAGCTCAG | GGGGTTTTCTGTCTTGGCAAC |
|  | *mfn1a* | TGTGGAGGAGAGAATGGTGA | GAGCAGAGGAAGAGGTGAGC |
|  | *mfn2* | CTCCAACTGCAGCCATCAA | TGGCACAGCTGAGAGAAGG |
|  | *nrf1* | ATGGCCCTCAACAGTGAAGC | CCGGAACTCCAGTTAACGCT |
|  | *β-actin* | GCAGAAGGAGATCACATCCCTGGC | CATTGCCGTCACCTTCACCGTTC |
|  | *18s rRNA* | CACTTGTCCCTCTAAGAAGTTGCA | GGTTGATTCCGATAACGAACGA |
|  | *nDNA* | ATGGGCTGGGCGATAAAATTGG | ACATGTGCATGTCGCTCCCAAA |
|  | *mtDNA* | CAAACACAAGCCTCGCCTGTTTAC | CACTGACTTGATGGGGGAGACAGT |
| Human  & Mouse | *nDNA* | TGCTGTCTCCATGTTTGATGTATCT | TCTCTGCTCCCCACCTCTAAGT |
|  | *mtDNA* | ATGGCCCACCATAATTACCC | CATTTTGGTTCTCAGGGTTTG |
