# Supplementary figures and images for: Smarca4 maintains mitochondrial homeostasis and energy metabolism during cardiac development
Source: Cell Mol Life Sci. 2026 Mar 7;83(1):167. doi: 10.1007/s00018-026-06168-3 (PMC13013801; doi:10.1007/s00018-026-06168-3)

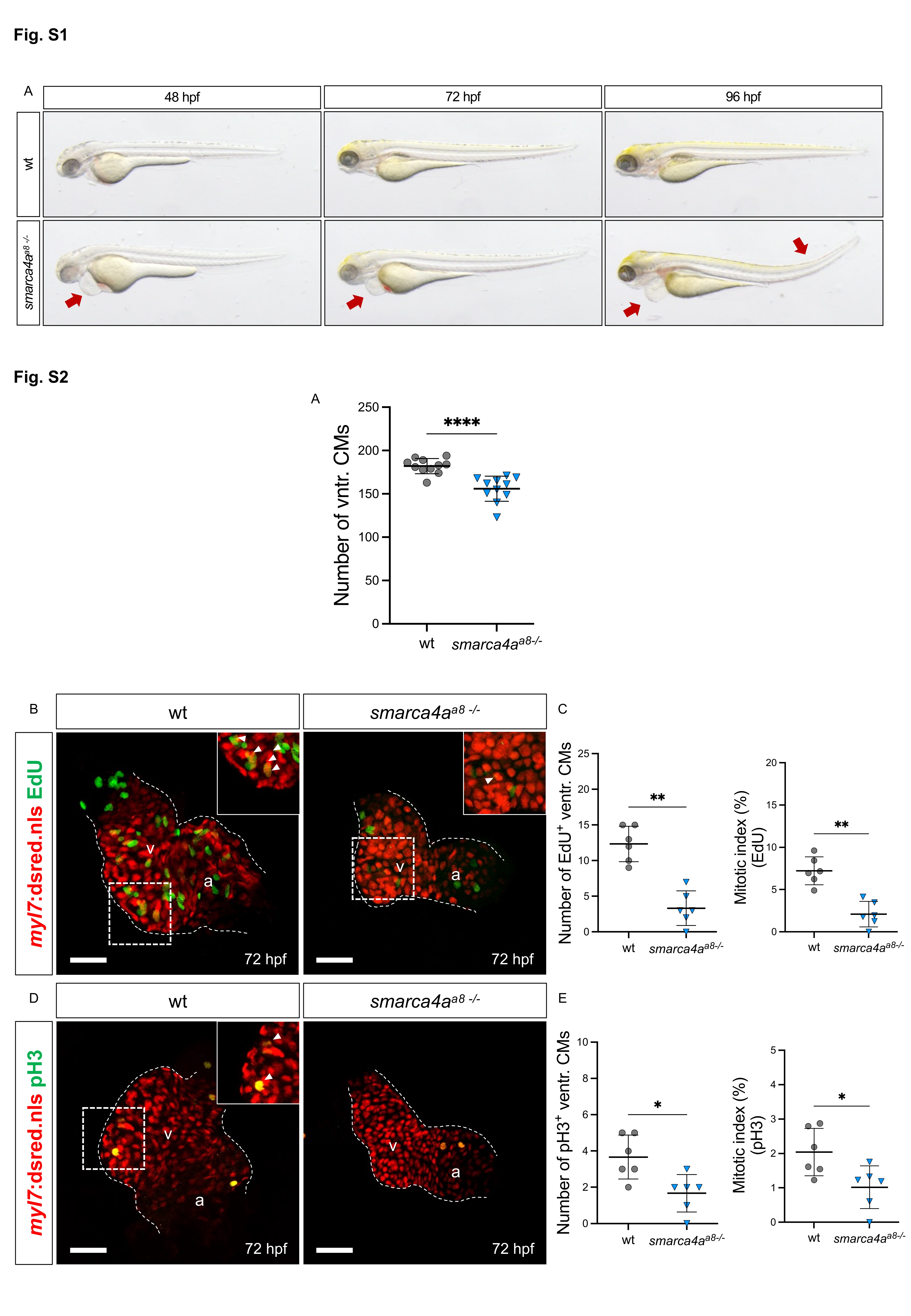

Supplement: Supplementary file 2 — Supplementary Material 2 Figure. S1: Mutation of smarca4 leads to cardiac and skeletal muscle phenotype during early development of zebrafish embryos. Figure. S2: Loss of Smarca4 decreases cardiomyocyte proliferation in developing heart of zebrafish. [file 18_2026_6168_MOESM2_ESM.jpg]

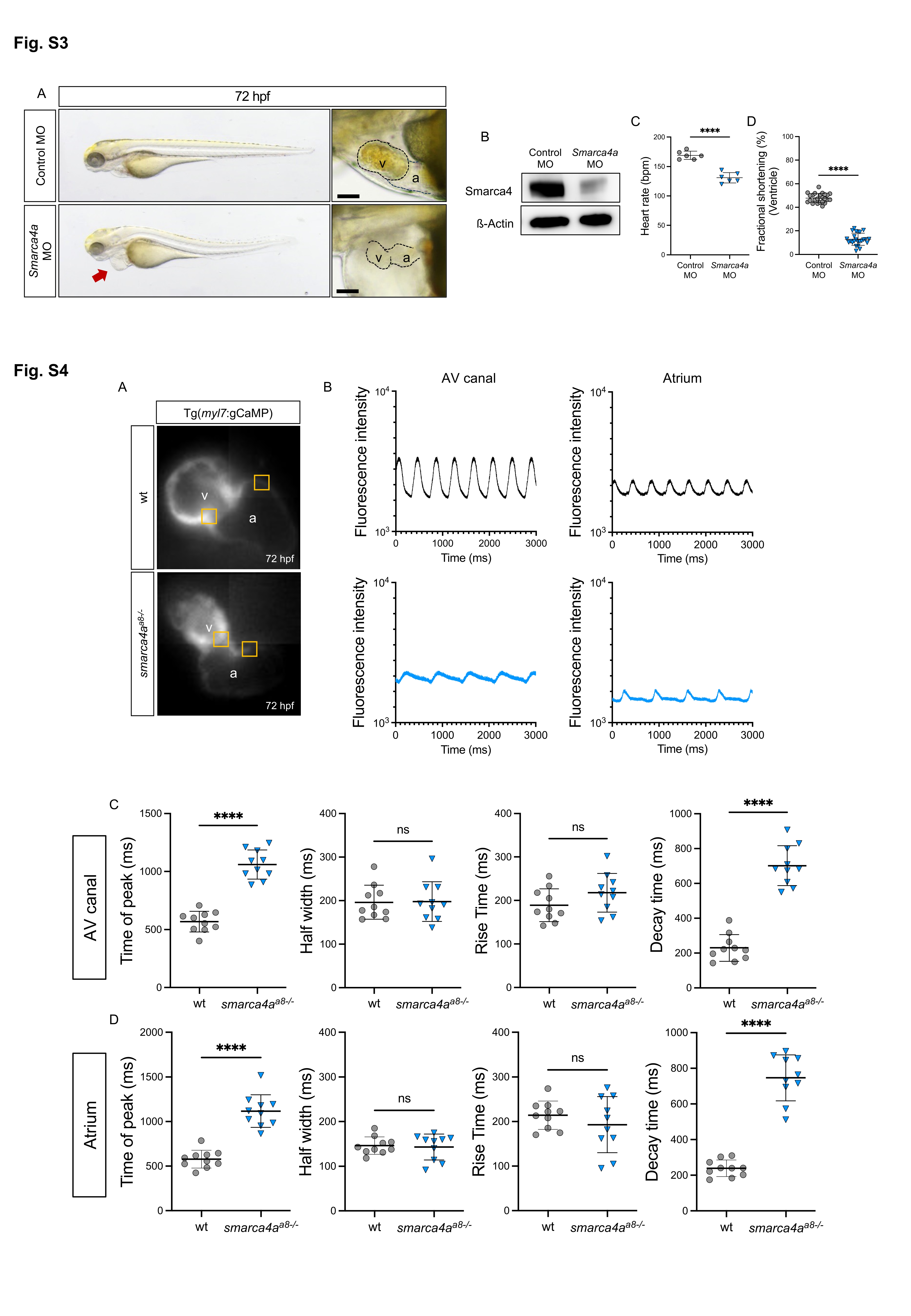

Supplement: Supplementary file 3 — Supplementary Material 3 Figure. S3: Morpholino-mediated Smarca4 inhibition recapitulates cardiac defects in zebrafish embryos. Figure. S4: Loss of smarca4 reduces calcium flux in zebrafish embryonic heart. [file 18_2026_6168_MOESM3_ESM.jpg]

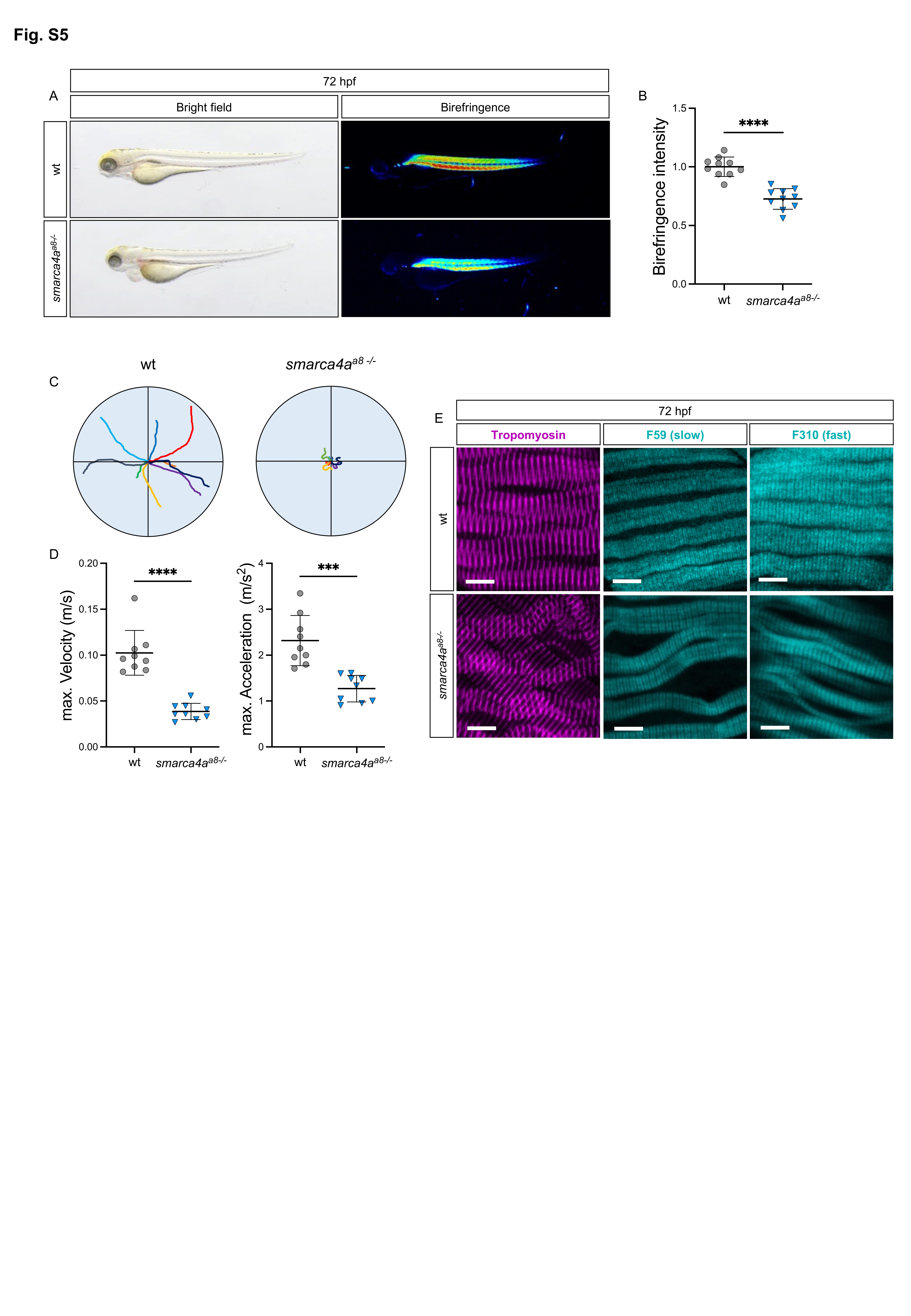

Supplement: Supplementary file 4 — Supplementary Material 4 Figure. S5: Ablated Smarca4 impairs skeletal muscle development and motility in zebrafish embryo. [file 18_2026_6168_MOESM4_ESM.jpg]

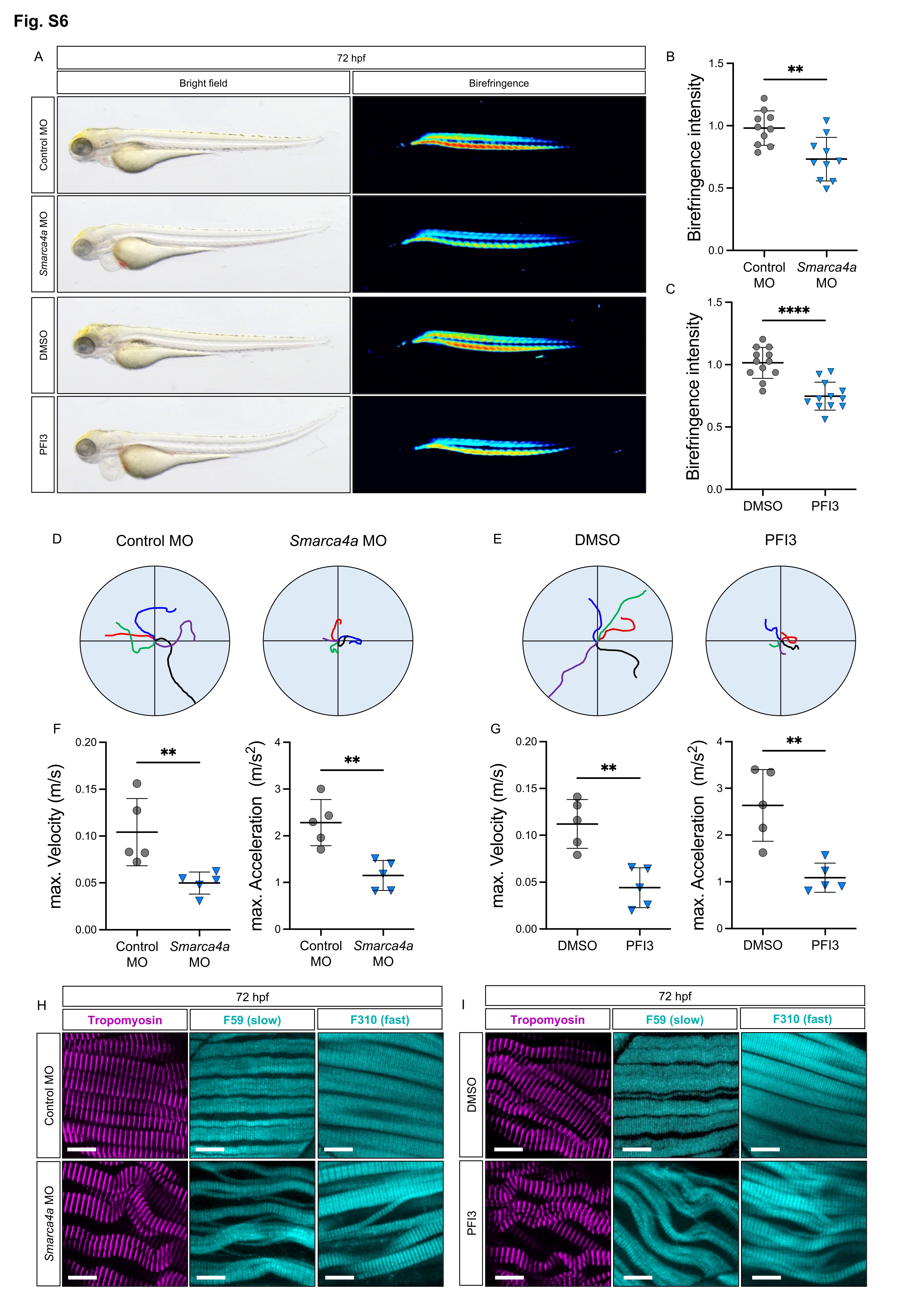

Supplement: Supplementary file 5 — Supplementary Material 5 Figure. S6: Inhibitory effect of smarca4a MO or PFI3 impairs skeletal muscle development and motility in zebrafish embryo. [file 18_2026_6168_MOESM5_ESM.jpg]

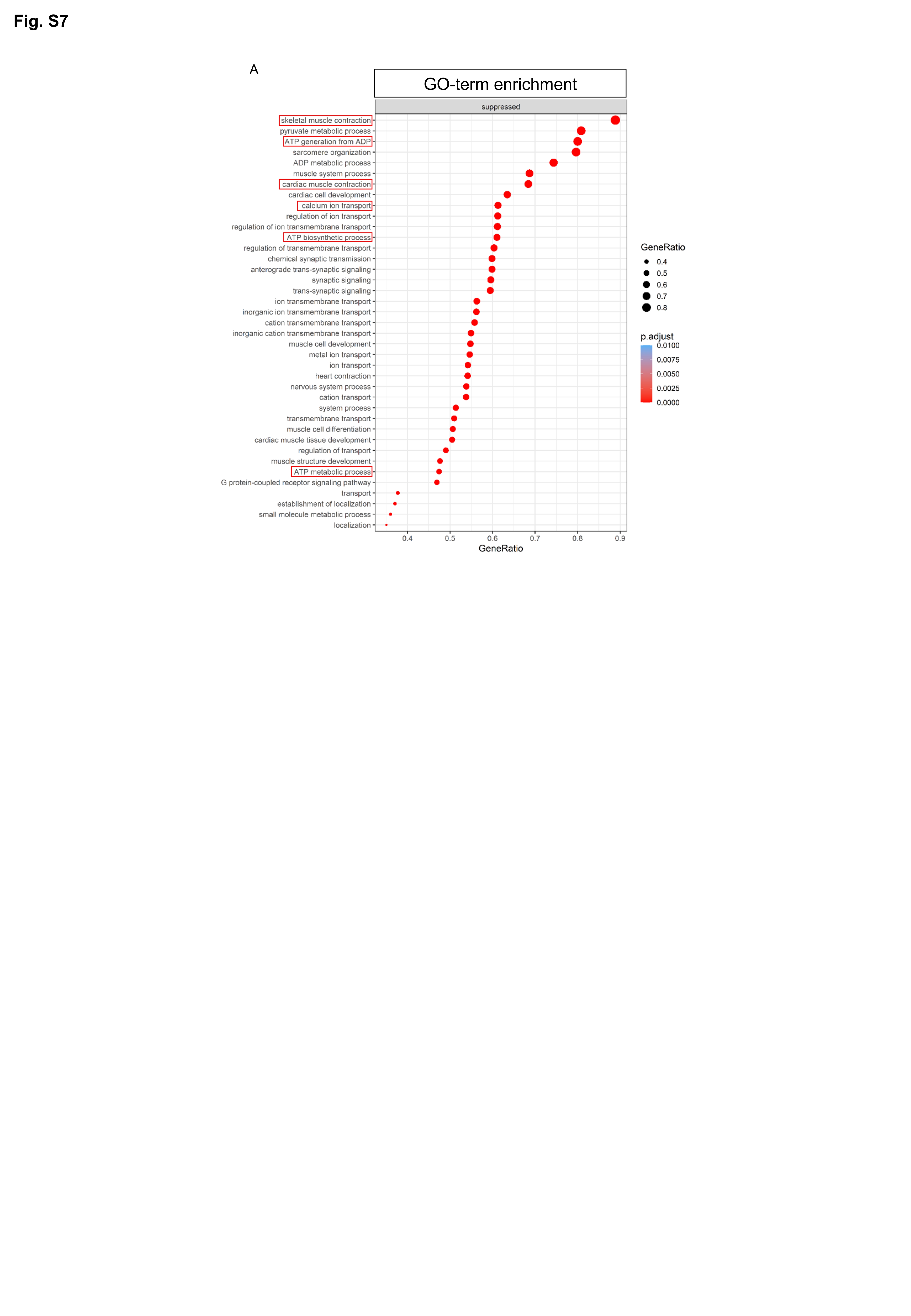

Supplement: Supplementary file 6 — Supplementary Material 6 Figure. S7: Heart-specific RNA-seq reveals suppressed gene ontology (GO) terms of muscle contraction and ATP production in smarca4aa8-/- hearts. [file 18_2026_6168_MOESM6_ESM.jpg]

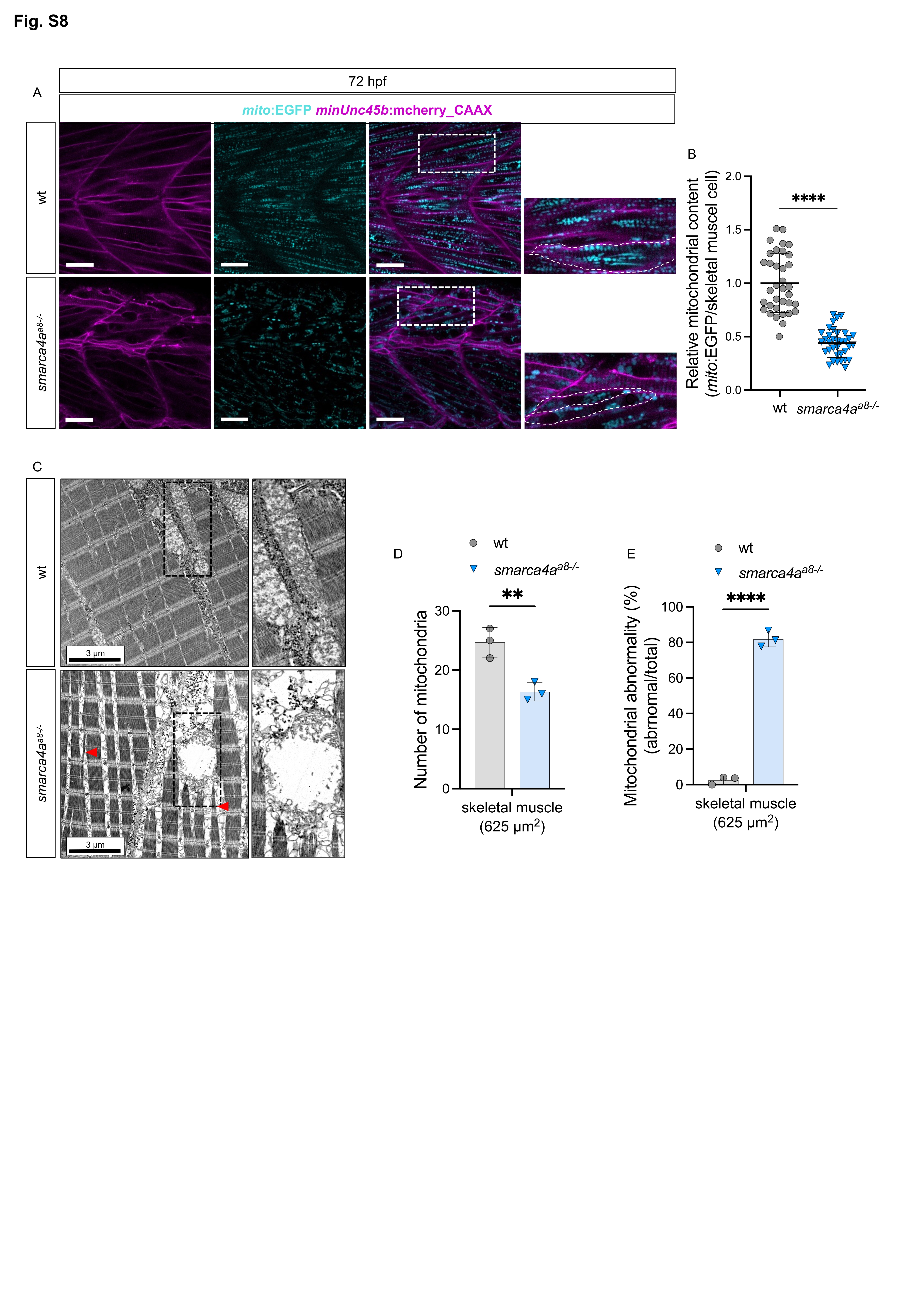

Supplement: Supplementary file 7 — Supplementary Material 7 Figure. S8: Mitochondria are impaired in skeletal muscle of smarca4aa8-/-. [file 18_2026_6168_MOESM7_ESM.jpg]

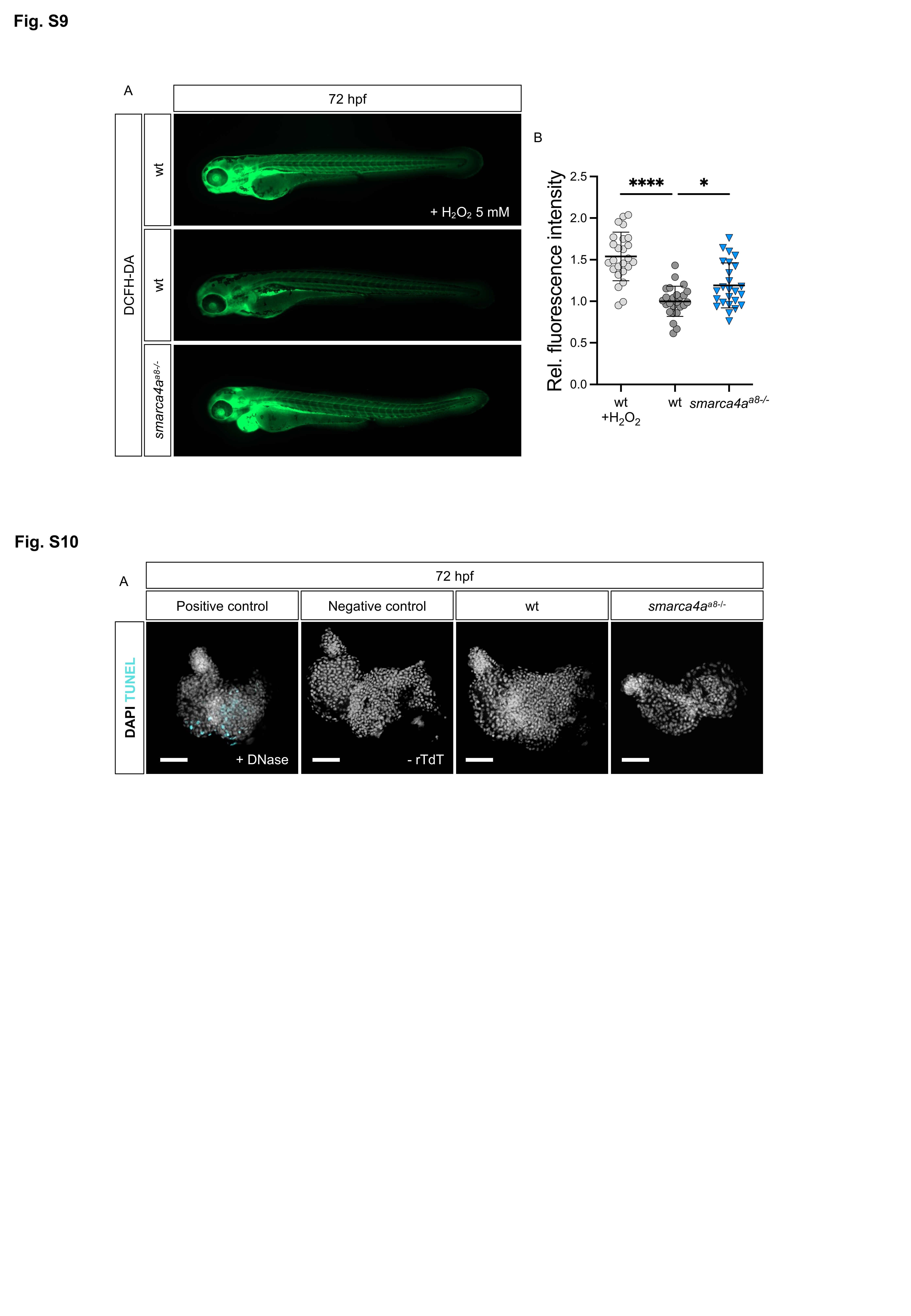

Supplement: Supplementary file 8 — Supplementary Material 8 Figure. S9: Reactive oxygen species (ROS) level is increased in smarca4aa8-/-. Figure. S10: Smarca4 mutation does not affect DNA damage induced apoptosis in zebrafish embryonic heart. [file 18_2026_6168_MOESM8_ESM.jpg]

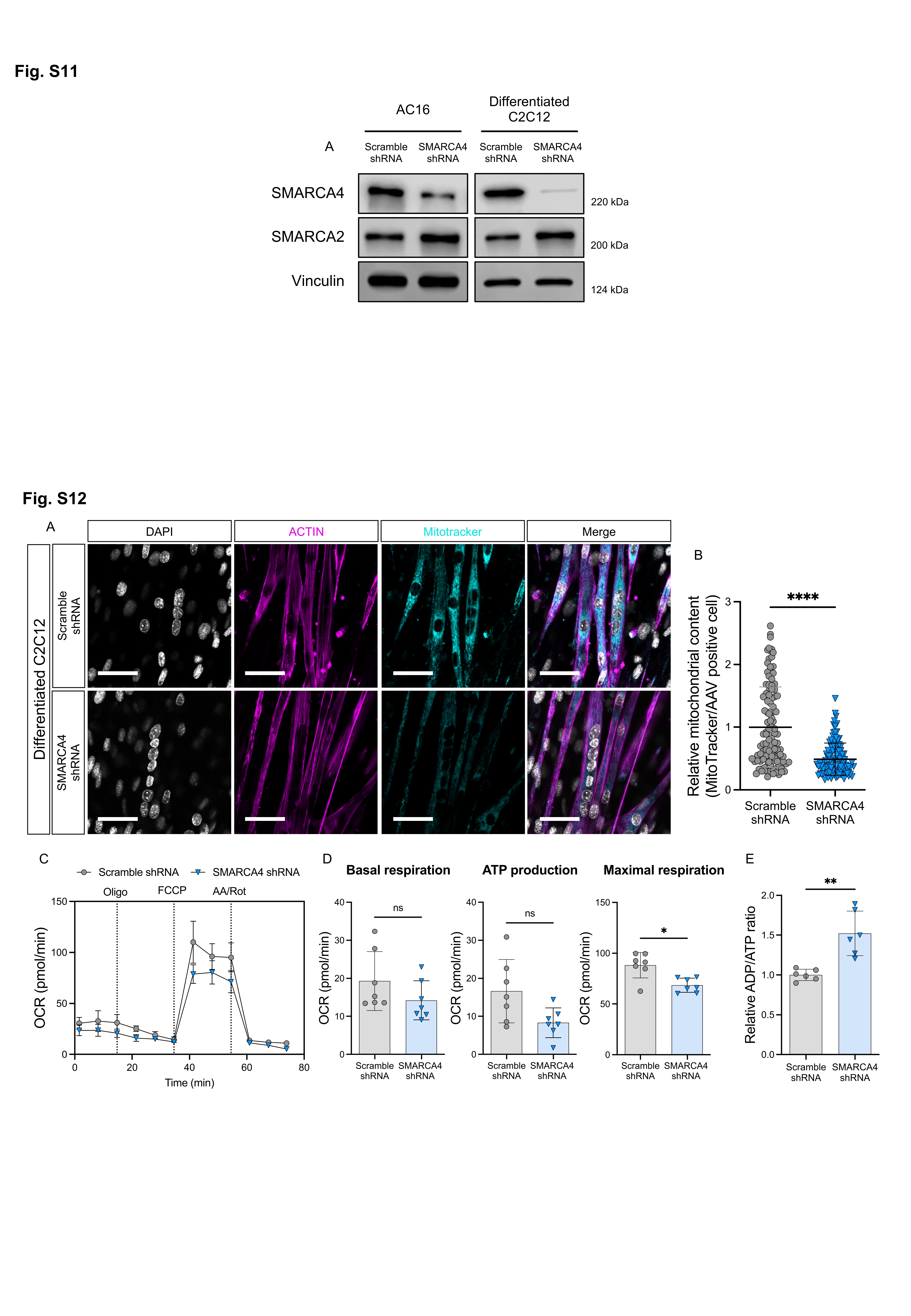

Supplement: Supplementary file 9 — Supplementary Material 9 Figure. S11: Efficient knockdown of SMARCA4 by AAV-SMARCA4 shRNA transduction in mammalian cardiomyocytes and skeletal muscle cells. Figure. S12: AAV-mediated SMARCA4 knockdown decreases mitochondrial respiration in mammalian skeletal muscle cells. [file 18_2026_6168_MOESM9_ESM.jpg]
